# Stretchable polymer composites with ultrahigh piezoelectric performance

**DOI:** 10.1093/nsr/nwad177

**Published:** 2023-06-22

**Authors:** Tongxiang Tang, Zhonghui Shen, Jian Wang, Shiqi Xu, Jiaxi Jiang, Jiahui Chang, Mengfan Guo, Youjun Fan, Yao Xiao, Zhihao Dong, Houbing Huang, Xiaoyan Li, Yihui Zhang, Danyang Wang, Long-Qing Chen, Ke Wang, Shujun Zhang, Ce-Wen Nan, Yang Shen

**Affiliations:** State Key Lab of New Ceramics and Fine Processing, School of Materials Science and Engineering, Tsinghua University, Beijing 100084, China; State Key Laboratory of Advanced Technology for Materials Synthesis and Processing, Center of Smart Materials and Devices, Wuhan University of Technology, Wuhan 430070, China; State Key Laboratory of Advanced Technology for Materials Synthesis and Processing, Center of Smart Materials and Devices, Wuhan University of Technology, Wuhan 430070, China; Advanced Research Institute of Multidisciplinary Science, Beijing Institute of Technology, Beijing 100081, China; Applied Mechanics Laboratory, Department of Engineering Mechanics, Tsinghua University, Beijing 100084, China; Applied Mechanics Laboratory, Department of Engineering Mechanics, Tsinghua University, Beijing 100084, China; Center for Flexible Electronics Technology, Tsinghua University, Beijing 100084, China; State Key Lab of New Ceramics and Fine Processing, School of Materials Science and Engineering, Tsinghua University, Beijing 100084, China; State Key Lab of New Ceramics and Fine Processing, School of Materials Science and Engineering, Tsinghua University, Beijing 100084, China; State Key Lab of New Ceramics and Fine Processing, School of Materials Science and Engineering, Tsinghua University, Beijing 100084, China; State Key Lab of New Ceramics and Fine Processing, School of Materials Science and Engineering, Tsinghua University, Beijing 100084, China; Advanced Research Institute of Multidisciplinary Science, Beijing Institute of Technology, Beijing 100081, China; Applied Mechanics Laboratory, Department of Engineering Mechanics, Tsinghua University, Beijing 100084, China; Applied Mechanics Laboratory, Department of Engineering Mechanics, Tsinghua University, Beijing 100084, China; Center for Flexible Electronics Technology, Tsinghua University, Beijing 100084, China; School of Materials Science and Engineering, University of New South Wales, Kensington, NSW 2052, Australia; Department of Materials Science and Engineering, The Pennsylvania State University, State College, PA 16802, USA; State Key Lab of New Ceramics and Fine Processing, School of Materials Science and Engineering, Tsinghua University, Beijing 100084, China; Institute for Superconducting and Electronic Materials, Australian Institute for Innovative Materials, University of Wollongong, Wollongong, NSW 2500, Australia; State Key Lab of New Ceramics and Fine Processing, School of Materials Science and Engineering, Tsinghua University, Beijing 100084, China; State Key Lab of New Ceramics and Fine Processing, School of Materials Science and Engineering, Tsinghua University, Beijing 100084, China; Center for Flexible Electronics Technology, Tsinghua University, Beijing 100084, China

**Keywords:** polymer composites, piezoelectric materials, structure design, flexible electronics

## Abstract

Flexible piezoelectric materials capable of withstanding large deformation play key roles in flexible electronics. Ferroelectric ceramics with a high piezoelectric coefficient are inherently brittle, whereas polar polymers exhibit a low piezoelectric coefficient. Here we report a highly stretchable/compressible piezoelectric composite composed of ferroelectric ceramic skeleton, elastomer matrix and relaxor ferroelectric-based hybrid at the ceramic/matrix interface as dielectric transition layers, exhibiting a giant piezoelectric coefficient of 250 picometers per volt, high electromechanical coupling factor *k_eff_* of 65%, ultralow acoustic impedance of 3MRyl and high cyclic stability under 50% compression strain. The superior flexibility and piezoelectric properties are attributed to the electric polarization and mechanical load transfer paths formed by the ceramic skeleton, and dielectric mismatch mitigation between ceramic fillers and elastomer matrix by the dielectric transition layer. The synergistic fusion of ultrahigh piezoelectric properties and superior flexibility in these polymer composites is expected to drive emerging applications in flexible smart electronics.

## Teaser

Through structure design and interface modifications, this work addressed the dielectric as well as mechanical mismatch between ceramic and polymer in composites, leading to soft composites with ultrahigh piezoelectric performances comparable to piezoelectric ceramics.

## INTRODUCTION

**Figure 1.**
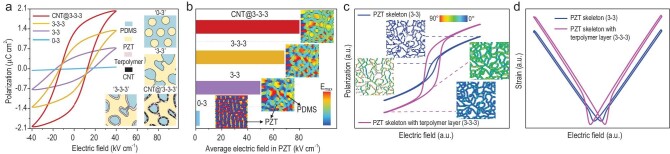
Design of ‘3–3–3’ type piezoelectric composites and simulation on local electric field distribution, domain structure and ferroelectric property in composites with different connectivity. (a) Polarization-electric field loop (*P-E* loop) of ‘0–3’, ‘3–3’ ‘3–3–3’ and CNT@‘3–3–3’ composites with composite schematic diagrams in the insets. (b) Phase-field simulation on local electric field distribution in polymer composites (inset images) with different structures at the same PZT volume fraction, and the simulated average electric fields in PZT skeleton (column chart). The applied electric field is set at 100 kV cm^−1^. The color bar represents the value of local electric field. (c) Phase-field simulation on polarization and domain structure of the PZT skeleton in ‘3–3’ and PZT skeleton with terpolymer layer in ‘3–3–3’ composites. The paraelectric elastomer matrix has a minor contribution to the dielectric and polarization properties which has been neglected in the simulation. The color bar represents the angle between the direction of the local polarization and external electric field. (d) Simulated strain-electric field loop (*S-E* loop) of the PZT skeleton in ‘3–3’ and ‘3–3–3’ composites. The electric field, polarization and strain in the simulations are all normalized.

Piezoelectric materials that convert mechanical energy into electrical energy (and vice versa) are at the heart of smart structures [1–3], which can perform both sensing and actuation functions. Recent developments in flexible electronics, such as wearable electronics and health monitoring systems, and soft robotics pose great challenges to flexible smart materials that can establish intimate, conformal contacts with complex curved surfaces while maintaining high levels of strain. High performance ferroelectric ceramics and single crystals have piezoelectric coefficient on the order of 1500 pC N^−1^ [4] and 4100 pC N^−1^ [5], respectively, far beyond that of other piezoelectric materials such as PVDF-based polar polymer [6,7], molecular perovskite solid solutions [8] or particle-filled polymer composites. However, the large product stiffness and density of ceramics leads to their inferior mechanical compliance and high acoustic impedance, hindering the efficient conversion between mechanical and electrical energies. To enable mechanically flexible materials with satisfactory piezoelectricity, polymer matrix composites have been intensively studied in constructing flexible and stretchable piezoelectric materials [9–13]. Piezoelectric composites have been pioneered by Newnham et.al. in the early 1970s [14], where piezoelectric ceramic fillers, such as lead zirconate titanate (PZT) [15], lead magnesium niobate-lead titanate (PMN-PT) [16] or barium titanate (BT) [17] have been introduced in the form of spheres or fibers into the polymer matrix to fabricate ‘0–3’ composite (‘0’means the ceramic filler is not connected in a continuous path in any dimension throughout the polymer matrix, while ‘3’ means the polymer matrix is connected in all of the 3 dimensions, as illustrated in the inset of Fig. 1a). By using BurPS (burn-off polymer spheres), a high content of ceramic filler is required to induce appreciable piezoelectric enhancement but results in a rigid composite ceramic. Polymer composites filled with arrays of aligned ceramic fibers belong to ‘1–3’ type composite, which may deliver a much higher piezoelectric coefficient. However, a high-volume fraction of ceramic fibers are still needed, and these composites exhibit only limited flexibility when bent over a certain angle.

The other major challenge for piezoelectric composites is the mismatching of electrical and mechanical properties between the ceramic fillers and polymer matrices, which, over the past decades, have severely hindered their synergistic fusion into stretchable piezoelectric composites with high piezoelectric properties. The large difference in dielectric constant between ferroelectric ceramic and polymer leads to a discontinuity in electric polarization at the filler/matrix interfaces and forms an electric double-layer that shields the dielectric response of ceramic fillers. The dielectric difference also leads to severe concentration of the local electric field in polymer matrix, hence substantially reducing the local electric field in the ferroelectric ceramic filler, thereby compromising the overall piezoelectric response of the composites. Analogous to the dielectric constant difference, the large difference in elastic modulus between ceramic and polymer also results in the concentration of local stress fields in the polymer matrix. Therefore, the mechanical load may not be efficiently transferred to the ceramic filler in order to induce a high piezoelectric response. Numerous strategies have been employed to establish transfer paths for both electric polarization and mechanical load in polymer matrix to provide robust composites, such as reinforcing polymer in 3 dimensions by low magnetic field [18], using freezing of ice to build complex composites [19], or bio-inspired assembly of nanoplatelets in a polymer matrix [20,21]. Yet all the strategies lead to a high volume fraction of ceramic fillers and severely compromise the mechanical compliance of ceramic/polymer composites [22–25].

Here we propose a combination approach to simultaneously address both dielectric and mechanical mismatch issues in order to achieve stretchable polymer composites with giant piezoelectric properties. We first assemble PZT particles into an interconnected skeleton inside the polydimethylsiloxane (PDMS) polymer matrix to form ‘3–3’ type composites, in which both PZT and PDMS form global networks as illustrated in Fig. 1a. The number of filler/matrix interface is thus minimized, where the reduced interfacial depolarization-field greatly improves the dielectric and ferroelectric properties of the composites at a very low filler volume fraction. The PZT skeleton also acts as effective load-transfer paths during the electromechanical coupling. We then use a thin relaxor ferroelectric polymer layer, polyvinylidene fluoride-trifluoroethylene-chlorofluoroethylene (P(VDF-TrFE-CFE), terpolymer) and carbon nanotubes (CNT), to modulate the local electric field distribution in the PZT skeletons (denoted as ‘3–3–3’ and CNT@‘3–3–3’ composites). We demonstrate that this combination approach gives rise to a record high piezoelectric property in stretchable polymer composites, e.g. d_33_* of 250 pm V^−1^ which is even comparable to commercial piezoelectric ceramics. Of particular importance, the composites can be stretched and compressed to a high elastic strain of 50% without sacrificing the piezoelectric properties, due to their low Young's modulus of <30 MPa. In addition, the composites exhibit a very low acoustic impedance of ∼3 MRayl, one order of magnitude lower than that of PZT ceramic, being beneficial to the design of ultrasonic transducers considering the complex acoustic impedance matching layers in ceramic-based transducers [26].

## RESULTS AND DISCUSSION

As shown in Fig. 1a, even with a high loading of ∼20 vol.% PZT, the ‘0–3’ composites only exhibit a paraelectric-like linear polarization loop due to the lack of global interconnection of PZT particles. For the ‘3–3’ composites, images of SEM and micro-computerized tomography (Micro-CT, Fig. S1) reveal the global-interconnection and uniform pore distribution of the PZT skeleton. In addition, the secondary open pores facilitate infiltration of PDMS into the PZT skeleton and lead to dense composites free of air voids. Owing to the electric polarization transfer path by the PZT skeleton, the ‘3–3’ composites with a low PZT volume fraction of ∼14 vol.% exhibit a typical ferroelectric hysteresis loop with a maximum polarization *P_m_* of 0.75 μC cm^−2^ and a coercive electric field of ∼15 kV cm^−1^ (Fig. 1a ‘3–3’).

We then address the dielectric mismatch between PZT (*ε_r_* of 1000) and PDMS (*ε_r_* of 4) by introducing P(VDF-TrFE-CFE) terpolymer with moderate dielectric constant (*ε_r_* of ∼50) as a dielectric transition layer at the PZT/PDMS interfaces. To further mitigate the dielectric mismatch, we incorporate CNTs into the terpolymer to increase dielectric constant of the dielectric transition layers. With ultralow volume fraction of terpolymer/CNT-terpolymer layer uniformly deposited on the PZT skeleton (Fig. S2), the secondary open pores on the PZT skeleton are preserved which ensures full infiltration of PDMS to form dense composites with 3–3–3 connectivity (Fig. S3). Albeit with their low volume fraction (∼1 vol.%), the CNT-terpolymer transition layer has important effects on the dielectric behavior of the corresponding composites. Unlike the ‘3–3’ composite whose dielectric constant increases monotonically with increasing temperature, the composite with CNT-terpolymer dielectric transition layer exhibits frequency dispersion and dielectric relaxation at near room temperature (Fig. S4), which is inherited from the terpolymer. This typical dielectric relaxation behavior suggests that the CNT-terpolymer dielectric transition layer also forms a continuous global network and endows the composite with intrinsic dielectric response of the terpolymer. Since PZT, terpolymer and PDMS are all connected in 3 dimensions, we define the composites with only terpolymer interface layers as ‘3–3–3’ composites, and those incorporated with CNTs in the terpolymer layer as CNT@‘3–3–3’ composites. As shown in Fig. 1a, with the same PZT volume fraction of ∼14 vol.%, robust ferroelectric polarization is readily observed from ‘3–3–3’ and CNT@‘3–3–3’ composites as indicated by *P_m_* (2 μC cm^−2^), being 50% higher than that of the ‘3–3–3’ composites (*P_m_* ∼1.3 μC cm^−2^). No significant increase in dielectric loss or electrical conductivity is observed for CNT@‘3–3–3’ composites owing to the ultralow volume content of CNT in composites (Figs S5 and S6).

The ferroelectric polarization of ‘3–3’ type composites at low PZT volume fractions is attributed to the continuous ferroelectric PZT connectivity [27]. The PZT skeleton eliminates most of the interface between PZT particles and PDMS matrix, hence suppressing the strong depolarization field at PZT/PDMS interfaces. To shed more light on the origin of the enhanced ferroelectric polarization in the ‘3–3–3’ and CNT@‘3–3–3’ composites, we use phase-field simulation to analyze the local electric field distribution and polarization evolution. For ‘3–3’ composites without dielectric transition layers, the PZT skeleton suppresses the depolarization field at the PZT/PDMS interfaces (Fig. S7a and b) and leads to an enhanced average local electric field of 50 kV cm^−1^ in PZT, much higher than the local electric field of 2 kV cm^−1^ in PZT of ‘0–3’ composite (Fig. 1b). However, the simulated average electric field in the PZT skeleton is still much lower than the applied electric field (100 kV cm^−1^). When considering a terpolymer dielectric transition layer, the average local electric field in PZT of ‘3–3–3’ composite increases to 68 kV cm^−1^ (Fig. S7d). Of particular interest is that, for the CNT@‘3–3–3’ composite, the incorporation of CNTs in the terpolymer layer substantially increases dielectric constant of the transition layer, and gives rise to a much enhanced average local electric field of ∼80 kV cm^−1^ in PZT, which is close to the applied electric field of 100 kV cm^−1^. Such increased local electric field in the PZT skeleton is conducive to enhance the overall ferroelectric polarization in CNT@‘3–3–3’ composite.

The results of phase-field simulation also indicate that the CNT-terpolymer dielectric transition layers complement the polarization path in open pores of the PZT skeleton, hence facilitating electric polarization switching of the PZT skeleton in CNT@‘3–3–3’ composite through interfacial coupling (Fig. S8, a–f). As shown in Fig. 1c, the simulated *P_m_* and remanent polarization (*P_r_*) of PZT skeleton coated with terpolymer layer are enhanced by ∼40% and 300% respectively. We calculate the electric polarization at different angles between the polarization vector and the poling direction (<001>) in the PZT skeleton. As shown in the insets of Fig. 1c, the polarization vectors are mostly aligned along the poling direction in the ‘3–3–3’ composite to form continuous polarization paths, giving rise to an increased overall ferroelectric polarization (Fig. S8g). Phase-field simulation (Fig. 1d) also indicates that the polarization orientation and domain evolution of the PZT skeleton with terpolymer layer lead to increased electric-field-induced strain, suggesting higher piezoelectric response of ‘3–3–3’ composites.

**Figure 2.**
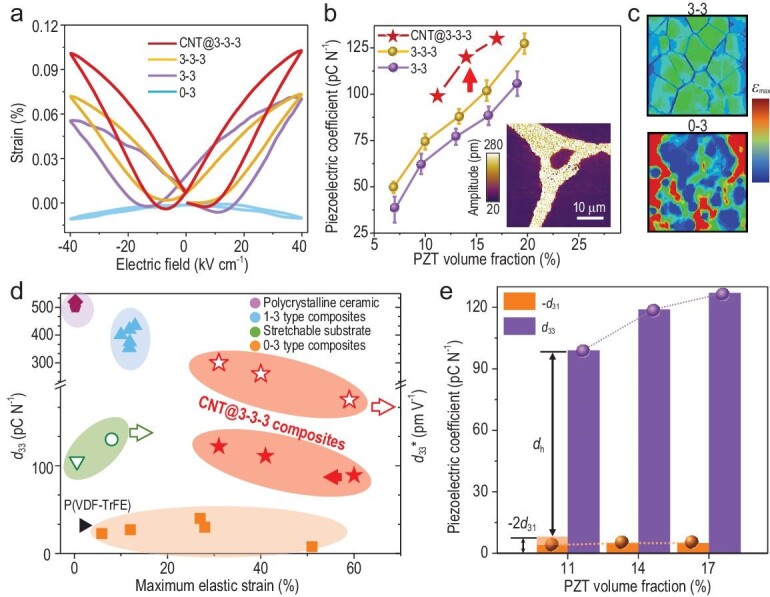
Ultrahigh piezoelectric properties of CNT@‘3–3–3’ composites. (a) *S-E* loop of polymer composites with different structures. The volume fraction of PZT in ‘3–3’, ‘3–3–3’ and CNT@‘3–3–3’ composites is ∼14 vol.%, while 20 vol.% PZT is introduced into ‘0–3’ composite. (b) Comparison of the piezoelectric charge coefficient of ‘3–3’, ‘3–3–3’ and CNT@‘3–3–3’ composites as a function of PZT volume fraction. The error bar represents experimental results of three different samples with the same PZT volume fraction. The inset picture in Fig. 2b shows the amplitude image of PFM test on ‘3–3’ composite, which demonstrates a local piezoelectric response of the PZT skeleton in polymer matrix. (c) Finite-element simulation of the maximum strain distribution in ‘0–3’ and ‘3–3’ type composites under uniaxial stretching (nominal strain = 5%). (d) Comparison of *d_33_* (solid symbols) and d_33_* (hollow symbols) at the maximum elastic strain for CNT@‘3–3–3’ composite (red stars) and other representative piezoelectric materials (*25, 28–33, 35*). (e) Variation of *d_33_* and *d_31_* of CNT@‘3–3–3’ composite as a function of PZT skeleton volume fraction. Hydrostatic piezoelectric coefficient *d_h_* follows the equation of *d_h_* = *d_33_* + *2d_31_* where the *d_31_* values of PZT and PZT-based composites are all negative.

We measure the electric-field-induced strain and observe typical butterfly strain-electric field (*S-E*) curves in all composites with PZT skeletons. As shown in Fig. 2a, with the same PZT volume fraction of ∼14 vol.%, the CNT@‘3–3–3’ composites deliver the highest strain of ∼0.1% among all the composites under an electric field of 40 kV cm^−1^, which is in line with the enhanced polarization as observed in *P-E* loops and the phase-field simulation. Yet, even with 20 vol.% of PZT particles, the ‘0–3’ composite still exhibits a slight negative electric-field-induced strain, a characteristic of an electrostrictive elastomer. We calculate the converse piezoelectric strain coefficient (d_33_*) from the *S-E* curves. The highest d_33_* of ∼250 pm V^−1^ is observed for CNT@‘3–3–3’ composite, which is 50% higher than that of ‘3–3–3’ composites (170 pm V^−1^).

Figure 2b shows piezoelectric charge coefficient (*d_33_*) measured by a quasi-static *d_33_*meter, where *d_33_* increases monotonically with increasing volume fraction of the PZT skeleton for all composites. The ‘3–3–3’ composites consistently exhibit higher *d_33_* than their ‘3–3’ counterparts due to the presence of a terpolymer transition layer, while the CNT-terpolymer transition layer induces the highest *d_33_* of 120 pC N^−1^ for CNT@‘3–3–3’ composites. Images of piezo-response force microscopy (PFM, inset of Fig. 2b) reveal a clear piezoelectric response and phase contrast in the PZT skeleton of CNT@‘3–3–3’ composites, while no obvious piezoelectric response can be observed in ‘0–3’ composites with uniformly distributed PZT particles. It is worth noting that the piezoelectric charge coefficient of CNT@‘3–3–3’ composite (*d_33_*∼120 pC N^−1^) is substantially lower than the converse piezoelectric strain coefficients (d_33_* ∼250 pm V^−1^). To elucidate this discrepancy, we perform a finite-element simulation on the stress/strain distribution in the composites. As shown in Fig. 2c, the severe local strain concentration in the polymer matrix of the ‘0–3’ composite is substantially relieved in the ‘3–3’ composite, and a distinct load-transfer path can be identified across the ‘3–3’ composites. However, due to the mechanical damping by PDMS matrix, the local strain within the PZT skeleton is only ∼20% of the overall strain of the composite (Fig. S9). The damped local strain in the PZT skeleton leads to a substantially lower *d_33_* of the composites than that of bulk ceramics, due to the smaller dynamic stress applied to the PZT skeleton during quasi-static measurement. In stark contrast, the CNT-terpolymer dielectric transition layer induces significantly enhanced local electric field (∼80 kV cm^−1^) in the PZT skeleton close to the overall applied electric field (∼100 kV cm^−1^), leading to a substantially increased electric-field-induced strain, i.e. the converse piezoelectric strain coefficient d_33_*. The intrinsic piezoelectric contribution obtained from Rayleigh analysis is ∼129 pm V^−1^ under electrical clamping conditions (Fig. S10) which is also consistent with piezoelectric charge coefficient measured by quasi-static methods.

The low volume fraction and hierarchical open pore structure of the PZT skeleton endow the CNT@‘3–3–3’ composites with excellent mechanical flexibility. A large elastic strain of 30%–60% is achieved (Fig. 2d) by tuning the volume fraction of the PZT skeleton. We compare the piezoelectric performance of our stretchable composites at maximum elastic strain with other representative piezoelectric materials. As shown in Fig. 2d, ferroelectric ceramics [28,29] exhibit the highest piezoelectric coefficients but can only sustain an elastic tensile strain below 0.2%. PZT or BT thin films deposited on stretchable substrate [30,31] can withstand an elastic tensile strain up to ∼10% but their piezoelectric performances are substantially compromised. Ferroelectric P(VDF-TrFE) [32] only exhibits a low *d_33_* of around −30 pC N^−1^ and small elastic tensile strain of <2%. With the anisotropic alignment of PZT fibers, the ‘1–3’ composite with comparable piezoelectric properties can be stretched up to ∼10% strain in the direction perpendicular to the longitudinal axis of the PZT fibers, but suffers from inferior bending flexibility [25,33,34]. On the other hand, the ‘0–3’ composites filled with a small amount of piezoelectric particles can be stretched to over 50% strain at the cost of very low piezoelectric coefficients [35]. In stark contrast, the CNT@‘3–3–3’ composites can deliver a high *d_33_* of 120 pC N^−1^ and d_33_* of 250 pm V^−1^ at elastic tensile strain of ∼30%. By tuning the volume fraction of the PZT skeleton, the CNT@‘3–3–3’ composites can even be stretched to ∼60% tensile strain and still provide high *d_33_* of 90 pC N^−1^ or d_33_* of 175 pm V^−1^.

In addition to high longitudinal piezoelectric coefficient (*d_3__3_*), the CNT@‘3–3–3’ composites also exhibit low transverse piezoelectric coefficient (*d_31_*). As shown in Fig. 2e, *d_3__3_*monotonically increases with increasing PZT volume fraction in CNT@‘3–3–3’ composites, while the consistently lower *d_31_* is insensitive to the PZT volume fraction. Results of the finite-element simulation indicate that PDMS with low Young's modulus acts as a mechanical damping medium and hinders the lateral load transfer in the composite (Fig. S11), leading to a low and composition-insensitive *d_31_*. The anisotropic piezoelectric response gives rise to high hydrostatic piezoelectric charge coefficient *d_h_* of ∼110 pC N^−1^ for the CNT@‘3–3–3’ composite (Fig. S12a), even higher than that of bulk PZT ceramics (*d_h_ ∼*100 pC N^−1^). The high *d_h_* and moderate *ε_r_* of CNT@‘3–3–3’ composite (*ε_r_* ∼97) also lead to an ultrahigh hydrostatic sensitivity coefficient (*d_h_ · g_h_*) of 14.3 pm^2^ N^−1^ (Fig. S12b), which is two orders of magnitude higher than the bulk PZT ceramics.

We summarize and compare the piezoelectric properties of our composites with four types of piezoelectric materials (Table 1). As seen, the CNT@‘3–3–3’ composite exhibits a high electromechanical coupling factor (*k_eff_*) of ∼0.65 (Fig. S12d), which is much higher than the thickness coupling *k_t_* and even comparable to the longitudinal coupling *k_33_* of PZT bulk ceramics. In addition, an ultralow acoustic impedance (3 MRayl) close to water is achieved in the CNT@‘3–3–3’ composite because of its ultralow bulk density of 1.9 g cm^−3^. The high coupling factor and ultralow acoustic impedance are expected to greatly benefit the application of ultrasonic transducers, whose sensitivity, power efficiency and bandwidth are closely associated with the coupling factor, while the low acoustic impedance is expected to simplify the design of acoustic impedance matching layers [26].

**Table 1.** Comparison of piezoelectric performances of different piezoelectric materials.

Piezoelectric materials	*d_33_* (pC N^−1^)	d_33_* (pm V^−1^)	*ε_r_*	Tanδ	*d_h_* (pC N^−1^)	*d_h_ g_h_* (pm^2^ N^−1^)	*k_eff_*	Z (MRayl)
PZT	474	550	2800	0.02	100	0.4	0.59	32
PVDF	−30	−31	10	0.05	6	6	0.146	3
0–3 composites	2	NA	12	0.002	2	0.03	NA	6
CNT@3–3–3 composites	120	250	97	0.02	110	14.3	∼0.65	3

Piezoelectric coefficients *d_33_* are measured by a Berlincourt *d_33_*-meter. The errors in the data are within 10%. The data for PZT is obtained from PZT ceramic sintered at the same temperature with PZT skeleton used to prepare the composites. *ε_r_* and Tanδ are measured at 1 kHz.

Taking advantage of the high piezoelectric coefficient, high electromechanical coupling factor and ultralow acoustic impedance of the CNT@‘3–3–3’ composite, we fabricate a prototype transducer without matching layer. The composite transducer exhibits an ultrahigh sensitivity and broad bandwidth of 40%, which is much improved compared to PZT- and P(VDF-TrFE)-based transducers fabricated under the same condition (Fig. S13). The output performance of the flexible composite-based transducer also remains stable even under 20% *in-situ* compression strain (Fig. S14).

**Figure 3.**
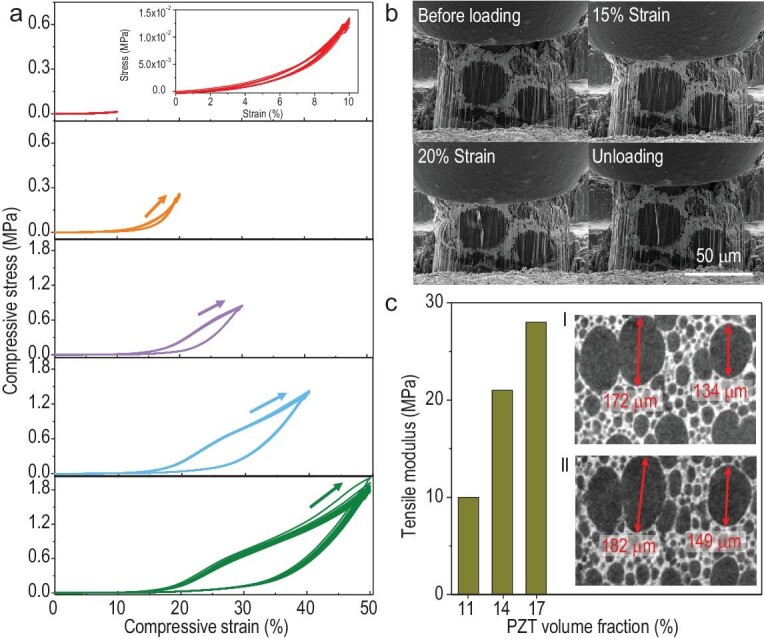
Super-compressibility and crack growth resistance of CNT@ ‘3–3–3’ composites. (a) Macroscopic compression cycling test with CNT@‘3–3–3’ composite under uniaxial compressive strain for 10 cycles. The composite thickness is about 1 mm. The arrow in each graph represents the loading stage of the first compression cycle. (b) *In-situ* SEM images of the microscopic compression cycling test for CNT@‘3–3–3’ composites. (c) Variation of tensile modulus as a function of PZT volume fraction in CNT@‘3–3–3’ composites. The inset Micro-CT pictures correspond to a composite with 16 vol.% of PZT I, at 0% strain state, and II after being uniaxially stretched to ∼12% strain.

To understand the superior flexibility and elucidate the origin of the mechanical compliance of the piezoelectric composites, we perform both macroscopic and *in-situ* microscopic uniaxial compression cycling measurements on the CNT@‘3–3–3’ composites. As shown in Fig. 3a, as the maximum cyclic strain gradually increases to 50%, the maximum stress rises rapidly from ∼0.3 MPa to ∼2 MPa, with an enhanced hysteresis observed in the cyclic compressive stress-strain curve, indicating a gradually increased mechanical loss of the composite during loading. This rapid rise in stress is induced by the compaction of the porous structure and increased friction between the rigid skeleton and the soft elastomer matrix [36,37]. Large compression deformation up to 50% can be fully recovered after unloading in the compressed state, indicating the PZT skeleton is intact during cyclic loading. For the composite compressed at 50% strain and aged for 24 h, we perform an additional 100 compression cycles and the composite is fully recovered (Fig. S15a).

We then perform *in-situ* cyclic uniaxial compression tests using SEM to observe changes in the structure of CNT@‘3–3–3’ composite during dynamic compression. PDMS matrix distributed in the PZT skeleton exhibits excellent resilience as the macroscopic deformation of the sample gradually increases (Supplementary Movies 1 & 2). Due to the hierarchical open pore structure, the secondary pores in the PZT skeleton wall provide the PDMS with free space to sustain considerable deformation. At compressive strain <15%, no visible cracks initiate in the composite and typical elastic stress-strain curves are observed, consistent with the macroscopic compression tests. At compressive strain of 20%, the first crack initiates and propagates in the PDMS matrix during the first compression cycle (Fig. 3b), accompanied by a drop in the stress-strain curve (Supplementary Movie 3). The PZT skeleton remains intact during compression, hinders the crack propagation in the matrix and confines the crack of PDMS inside one PZT pore in subsequent compression cycles (Fig. 3b, Supplementary Movie 4). The smooth stress-strain curve in the subsequent compression cycles also suggests no additional cracks are being developed in the composite (Fig. S15b). On the contrary, more cracks are initiated in the ‘0–3’ type composite under 20% compressive strain without constraint from the rigid PZT skeleton (Supplementary Movie 5). We also demonstrate a crack-free compression cycle in CNT@‘3–3–3’ composite during a 20% compression cycling test (Fig. S15c, Supplementary Movie 6) by reducing the PZT volume fraction from ∼14 vol.% to ∼10 vol.%, where a smaller compression modulus and less steep stress-strain curves are observed.

We also characterize the mechanical properties of the CNT@‘3–3–3’ composite under stretching conditions. As shown in Fig. 3c, the elastic tensile modulus of the composite gradually increases from 10 MPa to 28 MPa as the PZT volume fraction increases from 11 vol.% to 17 vol.%. We then conduct *in-situ* Micro-CT scanning on CNT@‘3–3–3’ composite with 17 vol.% of PZT under uniaxial stretch. The cross-section CT images reveal that the diameters of the corresponding two groups of pores increase from 172 μm and 134 μm at the initial 0% strain state (inset I of Fig. 3c), to 182 μm and 149 μm (inset II of Fig. 3c) at ∼12% tensile strain. The local elongation of the microporous structures is about 6% and 11%, respectively, which is on the same scale of the macroscopic tensile strain of 12%.

**Figure 4.**
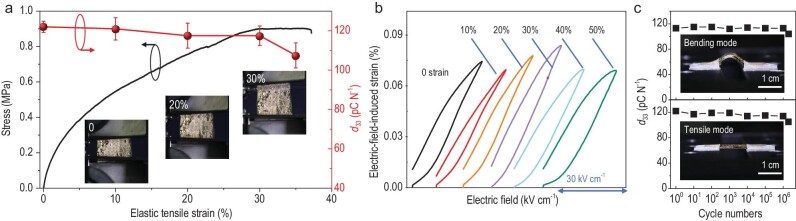
Stability of the piezoelectric properties of CNT@‘3–3–3’ composites under large elastic deformation and cycling tests. (a) Uniaxial tensile stress-strain curve of CNT@‘3–3–3’ composite and the piezoelectric charge coefficient variation of the reinstated composite after stretching to different strains. The error bar represents experiment results of three different points on the sample. The inset pictures correspond to composite stretching to 0%, 20% and 30% strains, respectively. (b) Electric-field-induced strain of CNT@‘3–3–3’ composite after 5 compression cycling tests as a function of maximum compressive strain. The samples used for characterization are poled under high voltage in advance. The maximum electric field in measurement is 30 kV cm^−1^. (c) Piezoelectric coefficient variation of the composite as a function of bending and stretching cycle number. The maximum cycling index is 10^6^. The bending radius is ∼5 mm while the tensile strain is ∼20%.

The collective uniform deformation of the PZT skeleton enables the CNT@‘3–3–3’ composite with stable piezoelectric properties under tensile strain, e.g. *d_33_* of 114 pC N^−1^ to be obtained after stretching the composite to 30% strain (Fig. 4a). The slight decrease in piezoelectric coefficient at tensile strain >30% is attributed to crack propagation in the composite. On the other hand, the unipolar *S-E* loop measured at 30 kV cm^−1^ shows that the electric-field-induced strain of CNT@‘3–3–3’ composite does not change substantially after 0%–50% strain compression cycles (Fig. 4b). The electric-field-induced strain increases slightly after a compression cycle of 40% strain, which may be related to the reduced damping effect of PDMS on the PZT skeleton. Also shown in Fig. 4c, the CNT@‘3–3–3’ composite exhibits excellent stability of the piezoelectric coefficient after 10^6^ cycles of bending or stretching tests, where the *d_33_* maintains the same value during the bending test and only decreases by ∼6% during the stretching test.

## CONCLUSION

In summary, we design and demonstrate highly stretchable/compressible polymer composites featuring ‘3–3–3’ connectivity with dielectric transition layer. The established electric polarization/load-transfer pathways and the mitigated local electric field concentration in the CNT@‘3–3–3’ composite lead to unprecedented properties that are not achievable in individual ceramic or polymer, including the high piezoelectric coefficients, high hydrostatic sensitivity, high electromechanical coupling factor and ultralow acoustic impedance close to water. These composites also exhibit high stability and reliability under giant elastic strain up to 50% and bending tests up to 2$\times $10^6^ cycles. Together with the facile fabrication process, the highly flexible composites with excellent piezoelectric properties provide a new design paradigm for smart structures and electromechanical devices, such as intravascular ultrasonic transducers, wearable self-powered devices with sensing and actuation functions, and soft robotics for intelligent functions.

## METHODS

The details of preparation and characterization of piezoelectric composites are shown in the online supplementary data.

## Supplementary Material

nwad177_Supplemental_Files
