# Supplementary material for: Stretchable polymer composites with ultrahigh piezoelectric performance
Source: Natl Sci Rev. 2023 Jun 22;10(8):nwad177. doi: 10.1093/nsr/nwad177 (PMC10359065; doi:10.1093/nsr/nwad177)
Supplement: nwad177_Supplemental_Files [file nwad177_supplemental_files.zip › Supplementary Data.pdf]

Supplementary data for&

Stretchable Polymer Composites with Ultrahigh Piezoelectric Performance

Tongxiang Tang[1], *Jian Wang*[2], *Zhonghui Shen*[2], *Shiqi Xu*[3], *Jiaxi Jiang*[4], *Jiahui Chang*[4,5], *Mengfan Guo*[1], *Youjun Fan*[1], *Yao Xiao*[1], *Zhihao Dong*[1], *Houbing Huang*[3], *Xiaoyan Li*[4], *Yihui Zhang*[4,5], *Ke Wang*[1], *Danyang Wang*[6], *Shujun Zhang*[7*], *Ce-Wen Nan*[1*], *Yang Shen*[1,5*]

Affiliations:

[1] State Key Lab of New Ceramics and Fine Processing, School of Materials Science and Engineering, Tsinghua University, Beijing 100084, China.

[2] State Key Laboratory of Advanced Technology for Materials Synthesis and Processing, Center of Smart Materials and Devices, Wuhan University of Technology, Wuhan 430070, China.

[3] Advanced Research Institute of Multidisciplinary Science, Beijing Institute of Technology, Beijing 100081, China.

[4] Applied Mechanics Laboratory, Department of Engineering Mechanics, Tsinghua University, Beijing 100084, China

[5] Center for Flexible Electronics Technology, Tsinghua University, Beijing 100084, China

[6] School of Materials Science and Engineering, University of New South Wales, Kensington, NSW 2052, Australia.

[7] ISEM, Australian Institute for Innovative Materials, University of Wollongong, Wollongong, NSW 2500, Australia.

Supplementary note 1 Materials and methods

Fabrication of piezoelectric composites

The lead zirconate titanate (PZT) powder was obtained from Qingfeng company, Jiaxing, Zhejiang. PDMS-184 silicon elastomer was purchased from the Dow Chemical company. The P(VDF-TrFE-CFE) (terpolymer) and P(VDF-TrFE) (copolymer) were obtained from Arkema and used as-received. Carbon nanotubes (CNTs) were obtained from XF Nano, Nanjing. All the chemicals were purchased from China National Chemicals Corp. Ltd. if not specified otherwise.

Porous ceramic skeleton based on PZT powder was fabricated via a surfactant-assisted foaming method followed by a sintering process [1]. The as-obtained ceramic powder was mixed with deionized water and treated with cell crusher for 60 min to ensure the well dispersion of particles in slurry. Then 0.05-0.1wt% of lauryl sodium sulfate surfactant was added into the as-prepared ceramic slurry, and appropriate amount of hydrochloric acid was dropwise added into the slurry to adjust the pH of the solution system. Small amount of carbon black was co-foamed with PZT powder [2, 3]. The slurry solution was stirred at room temperature and the stirring speed was fixed at 400 r min^{-1} for 60 min. A foaming process was conducted by high-speed mechanical stirring of ceramic slurry for 30 min at 1500-2000 r min^{-1}. The as-prepared wet foam was then dried at room temperature for 48 h and sintered between 1150°C and 1250°C for 120 min with fixed heating rate of 5°C min^{-1}, to form densified ceramic skeleton. The porosity of the ceramic skeleton which determines the final volume fraction of ceramic in polymer matrix was carefully controlled by adjusting the ratio of water to ceramic powder in the slurry and the amount of surfactant.

For '3-3'composites, the ceramic skeleton was composited with PDMS by immersing the ceramic skeleton in elastomer fluid and degassed in vacuum chamber at room temperature with the vacuum degree kept at ~5 Pa for 12h, followed by a heat curing

process at 110°C for 60 min.

For '3-3-3' type composites, the ceramic skeleton was first immersed into N, N-dimethylformamide (DMF) solution of terpolymer with controlled concentration of 7/15 w/v at room temperature and underwent an ultrasonication process with fixed power of 45% for 30 min. To obtain CNT@'3-3-3' composites, 0.0005-0.005 g CNTs were mixed with terpolymer solution before ultrasonication. The PZT skeleton was then immersed in the dilute solution of terpolymer and CNTs. A continuous CNT-terpolymer layer with thickness of < 100 nm was then deposited on the interconnected PZT skeleton. The thermogravimetric analysis indicates that the content of CNT-terpolymer layer in the final composites is ~ 1-2 vol%. Then the sample was dried at 130°C for 4 h, followed by the same compositing method as '3-3' type composites. The obtained composites were cut into cuboid samples and sputtered with gold (Au) electrodes for dielectric and piezoelectric property characterizations.

'0-3' type composites were prepared by directly mixing ceramic powder with PDMS followed by curing process.

Micromorphology and structural characterization

The Scanning Electron Microscopy (SEM) experiments were carried out using commercial GEMINISEM500, Zeiss scanning electron microscope.

Microscopic piezoelectric response of composites was measured by piezo-response force microscopy (PFM) using commercial scanning probe microscope (Asylum, MFP-3D). The Si cantilevers with conducting Pt/Ir coating layer and spring constant of 2.8 N m^{-1} (NanoSensors-PPP-EFM-50) were used. For PFM imaging, vector mode was used where tips were modulated at ~350 kHz for Out-of-plane imaging and ~700 kHz for In-plane imaging, with the AC voltage set at 1-1.5V. During the image capture, the scan rate was set at 0.6-1 Hz to ensure a high-quality piezoelectric response imaging.

Characterizations of dielectric and piezoelectric properties

Dielectric frequency spectroscopy and temperature-dependent broadband dielectric spectroscopy were performed on a precision impedance analyzer (Concept 40, Novo control) equipped with temperature controller (Quatro Cryosystem, Novo control). The perturbation voltage was set at 1 V. The piezoelectric charge coefficients were measured by a quasi-static d_{33}-meter (ZJ-3A). Electric-field-induced-strain (or *S*-*E* loop) and polarization loop (*P*-*E* loop) were measured by TF-analyzer 2000. The effective electromechanical coupling factor was calculated based on the resonance and anti-resonance frequencies measured by impedance analyzer (Concept 40, Novo control). The composite samples for dielectric and piezoelectric characterizations (P-E loop, S-E loop, quasi-static piezoelectric measurement and dielectric spectrum measurement) are ~1 mm in thickness and sputtered with circular electrodes about 5mm in diameter.

Mechanical property characterization

Uniaxial tensile and compressive tests of the composites were conducted using a Shimadzu AG-X universal tester (Shimadzu Corporation, Japan) at room temperature. The composites were uniaxially stretched to rupture. The cuboid samples for macroscopic compression tests (Fig. 3a) are 1 mm in thickness and 20 mm in side length. The cuboid samples for uniaxial tensile test (Fig. 4a) are 1 mm in thickness, and the clamped length is 10 mm with both ends of the composites encapsulated with epoxy plates in order to avoid failure at the clamping area. The cuboid samples for bending and stretching cycles are ~0.8 mm in thickness. The stress-strain curves were automatically recorded by a software connected with universal tester. *In-situ* uniaxial tensile tests and computerized tomography (Micro-CT) were performed via Micro-CT system of Xradia 620 Versa, Zeiss. *In-situ* cycling compressive tests on composites were conducted by Hysitron PI 85 PicoIndenter inside the SEM (Quanta FEG 450).

The as-fabricated CNT@’3-3-3’ composite is etched by focused ion beam (FIB) into cylinders with a diameter of about 70-100 μm and a height of 70-80 μm. Uniaxial compressive stress is applied to the composite by a conical nano-indenter with a 100-μm-diameter flat tip.

Fabrication and measurement of the prototype ultrasonic transducers

The prototype ultrasonic transducers were fabricated by attaching the poled piezoelectric composite to the acoustic backing layer by glue. The piezoelectric composite is ~ 200μm in thickness. The acoustic backing layer was prepared by mixing the epoxy resin with tungsten powder (W, 25 μm) and alumina (Al_2O_3, 50 μm) followed by a curing process. The mixture was cured at 40°C for 12 h to obtain the backing layer with thickness about 5 mm. The performance of the ultrasonic transducers was analyzed by detecting the received signals. In direct-contact mode, the transducer was placed in the coupling agent and in direct contact with a pulser/receiver (model 5072PR) with -35 dB amplifier gain. In underwater mode, the transducer was placed in a water tank and the ultrasonic probe was placed 1 mm away from the front of the transducer. The pulse signals and frequency-domain responses were obtained from the oscilloscope.

Phase-field simulations

In the phase-field simulation, the spatial temporal evolution of the polarization is described by the time-dependent Ginzburg-Landau equation (TDGL),

$$\frac{\partial P_i(\boldsymbol{r},t)}{\partial t} = -L\frac{\delta F}{\delta P_i(\boldsymbol{r},t)}, \quad (i = 1,2,3) \tag{1}$$

where L is the kinetic coefficient, and F is the total free energy of the system, which is expressed as,

$$F = \iiint (f_{bulk} + f_{elastic} + f_{electric} + f_{grad}) dV \tag{2}$$

where V is the system volume. The bulk energy density f_{bulk} can be calculated by,

$$\begin{aligned} f_{\text{bulk}} = {} & \alpha_1\left(P_1^2+P_2^2+P_3^2\right)+\alpha_{11}\left(P_1^4+P_2^4+P_3^4\right) \\ & +\alpha_{12}\left(P_1^2P_2^2+P_1^2P_3^2+P_2^2P_3^2\right)+\alpha_{112}\left[P_1^4\left(P_2^2+P_3^2\right)\right. \\ & \left.+P_2^4\left(P_1^2+P_3^2\right)+P_3^4\left(P_1^2+P_2^2\right)\right]+\alpha_{111}\left(P_1^6+P_2^6+P_3^6\right) \\ & +\alpha_{123}P_1^2P_2^2P_3^2 \end{aligned} \tag{3}$$

where P_1, P_2, P_3 are polarization components. α_1, α_{11}, α_{12}, α_{111}, α_{112} and α_{123} are Landau coefficients. The elastic energy density can be expressed as,

$$f_{elastic} = \frac{1}{2} c_{ijkl} e_{ij} e_{kl} = \frac{1}{2} c_{ijkl} (\varepsilon_{ij} - \varepsilon_{ij}^0)(\varepsilon_{kl} - \varepsilon_{kl}^0) \tag{4}$$

where c_{ijkl} is the elastic stiffness constant, e_{ij} is the elastic strain, ε_{ij} is the total strain, and ε_{ij}^0 is the eigenstrain (electrostrictive stress-free strain). Using the cubic phase as the reference, ε_{ij}^0 can be calculated by $\varepsilon_{ij}^0 = Q_{ijkl} P_k P_l$, where Q_{ijkl} is the electrostrictive coefficient. The gradient energy density can be expressed as,

$$f_{grad} = \frac{1}{2} G_{ijkl} \frac{\partial P_i}{\partial r_j} \frac{\partial P_k}{\partial r_l} \tag{5}$$

where G_{ijkl} is the gradient energy coefficient. The electrostatic energy density is expressed as,

$$f_{electric} = -\frac{1}{2} \varepsilon_0 K_{ij}^b E_i E_j - E_i P_i \tag{6}$$

where K_{ij}^b is the background dielectric constant and E_{i} is the electric field, which can be calculated with the equation,

$$E_i = -\frac{\partial \varphi}{\partial r_i} \tag{7}$$

The electric potential φ can be obtained by solving the electrostatic equilibrium equation,

$$\varepsilon_0 K_{ij}^b \frac{\partial^2 \varphi}{\partial r_i \partial r_j} = - \frac{\partial P_i}{\partial r_i} \tag{8}$$

Equations are numerically solved by semi-implicit Fourier-spectral method [4]. The simulation size is 256 Δx × 256 Δx, and the grid space in real space is Δx = 1.0 nm. When evaluating the change of local electric field due to the mismatch of polarization of different phases, the average electric field of different phase is introduced. In the simulation of polarization-electric field loop (*P*-*E* loop) and Strain-electric field loop (*S*-*E* loop), the applied electric field is 100 MV m^{-1}. In the simulation of local electric field distribution, the dielectric constant of PDMS, PZT and terpolymer are fixed at 4, 1000 and 50 when solving the Poisson's equation. Then, more possible systems are considered to study the trend of polarization mismatch.

Finite-element simulation

The finite-element analyses (FEA) were performed employing commercial software ABAQUS (SIMULIA, Providence RI) to calculate the deformations of '0-3' and '3-3' composites. Ten-node quadratic tetrahedron bricks were adopted with refined meshes to ensure computational accuracy. Linear elastic constitutive relationships of ceramic were used to simplify the simulation. All the ceramic elements are embedded into PDMS matrix using embedding technique available in ABAQUS.

Supplementary note 2 Morphology characterization of composites

This section analyzes the micro-morphology of PZT skeleton and piezoelectric composites in order to demonstrate the uniform deposition of terpolymer layer on PZT skeleton and the formation of dense composites without air voids.

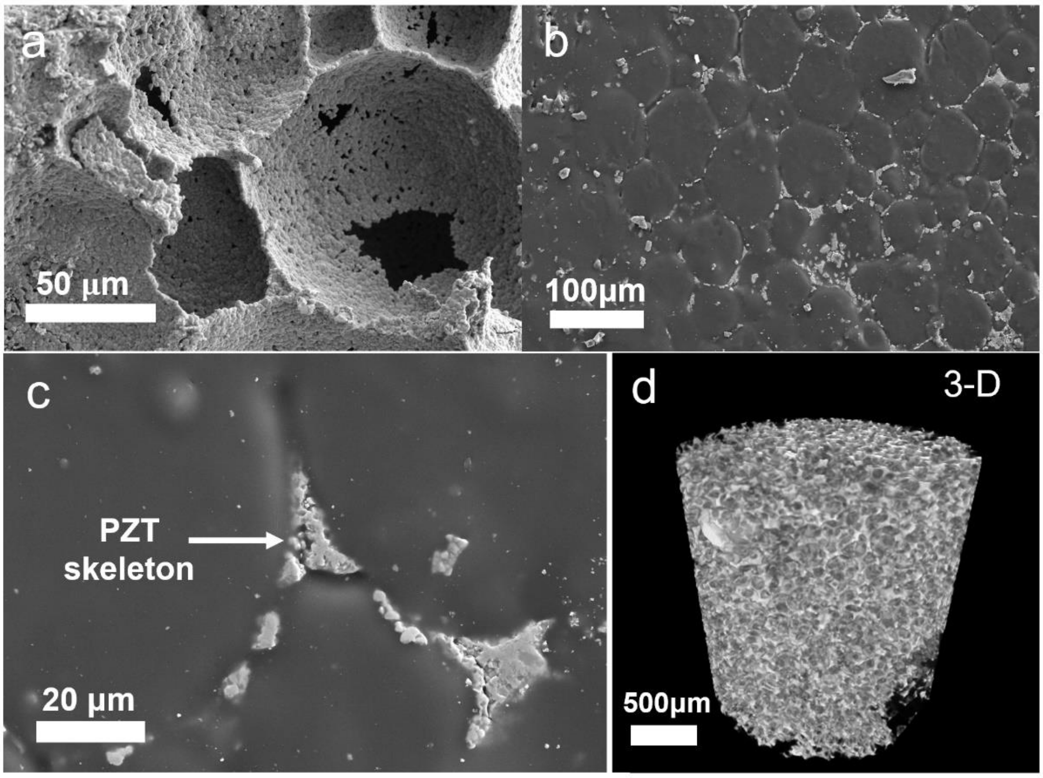

Fig. S1. Microstructure of PZT skeleton and as-fabricated composites. (**a**) SEM image of PZT skeleton sintered at 1200°C. Mesopores with diameter ranging from several microns to tens of microns are distributed on the skeleton. (**b**), (**c**) Cross-section SEM images of '3-3' composites. The PZT skeleton embedded in the polymer matrix is highlighted by the white arrow in the figure. (**d**) Micro-CT image of '3-3' composite.

As can be seen from SEM images in Fig. S1a-S1c, the porous structure enables successful infiltration of PDMS into PZT skeleton and uniform distribution of open pores is verified via Micro-CT characterization (Fig. S1d). The PZT skeleton forms interconnected structure with the thickness of the pore walls as small as 1-2 µm in the

as-fabricated ‘3-3’ composites which is essential for the mechanical compliance of the composites.

A thin layer of terpolymer with thickness around several tens of nanometers is uniformly coated on the PZT skeleton (Fig. S2a and b) which serves as the skeleton of ‘3-3-3’ composites. To fabricate CNT@‘3-3-3’ composites, PZT skeleton is coated with CNT-terpolymer blends and we demonstrate that the CNT-terpolymer blends is also uniformly deposited on the PZT skeleton with CNTs embedded in the terpolymer layer (Fig. S2c and d).

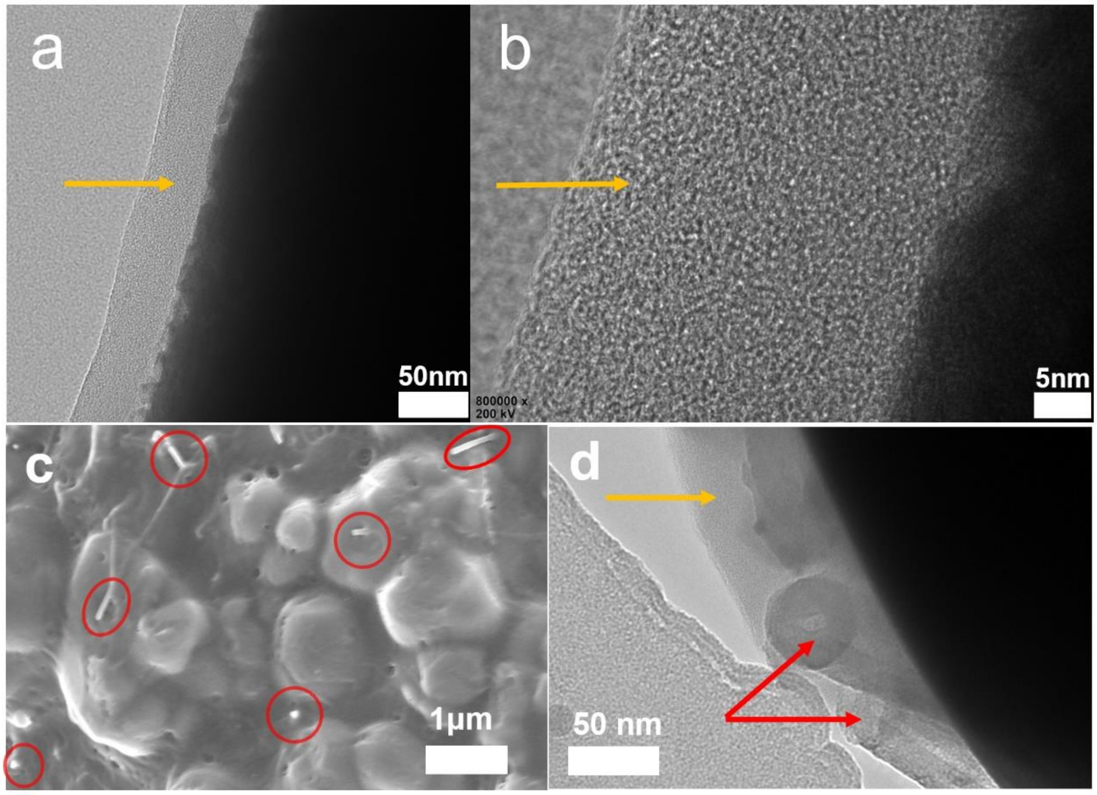

Fig. S2. Microstructure of PZT skeleton coated with terpolymer/CNT-terpolymer layer. (**a**), (**b**) TEM images of PZT skeleton coated by terpolymer layer (corresponding to ‘3-3-3’ composites). SEM (**c**) and TEM (**d**) images of PZT skeleton coated with CNT-terpolymer layer (corresponding to CNT@‘3-3-3’ composites).

It is of great significance that the deposition of terpolymer/CNT-terpolymer layer on the PZT skeleton doesn’t block the open porous structure which ensures the formation of dense polymer composites with 3-3-3 connectivity after infiltration of PDMS

matrix. As can be seen from Fig. S3, the terpolymer layer is selectively deposited on the PZT skeleton where large open pores are not covered. Consequently, the open porous structure facilitates the infiltration of PDMS into PZT skeleton which leads to dense composite without air voids in composites with 3-3-3 connectivity (Fig. S3d).

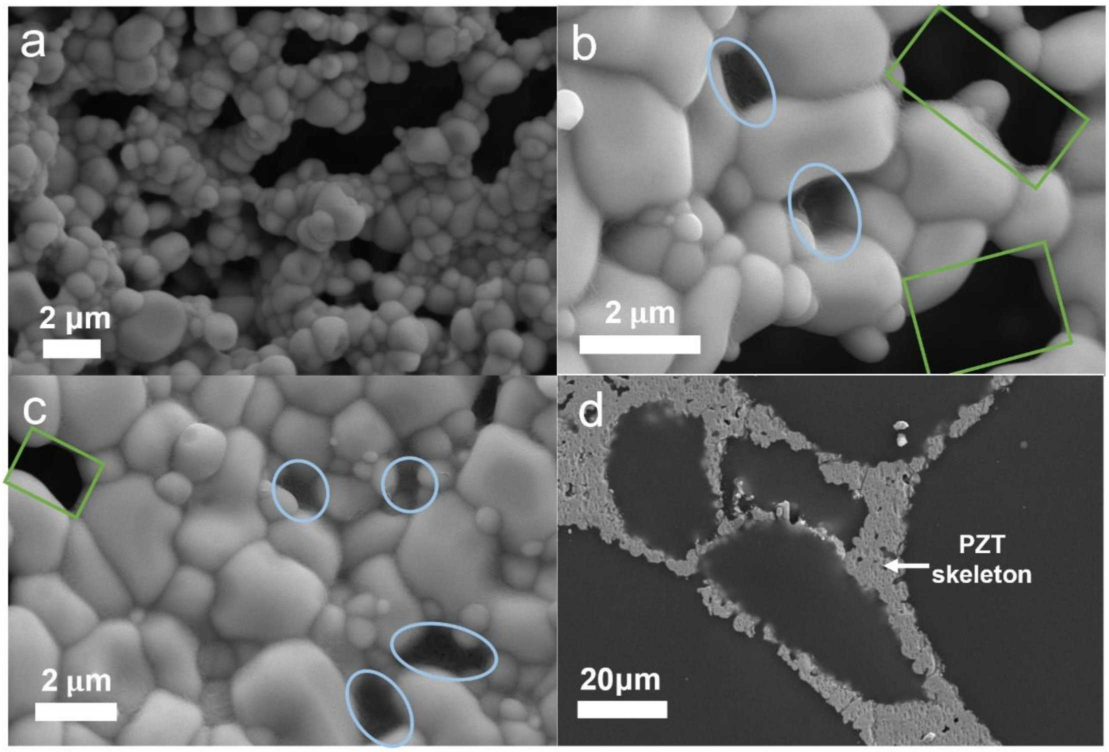

Fig. S3. SEM images of (**a**)-(**c**) PZT skeleton coated with terpolymer layer and (**d**) the cross-section area of the final composite filled with PDMS. The region circled by blue and green lines correspond to small open pores covered by terpolymer layer and relatively large open pores without being covered by terpolymer layer.

Supplementary note 3 Dielectric properties of composites

Dielectric response of composites with different connectivity

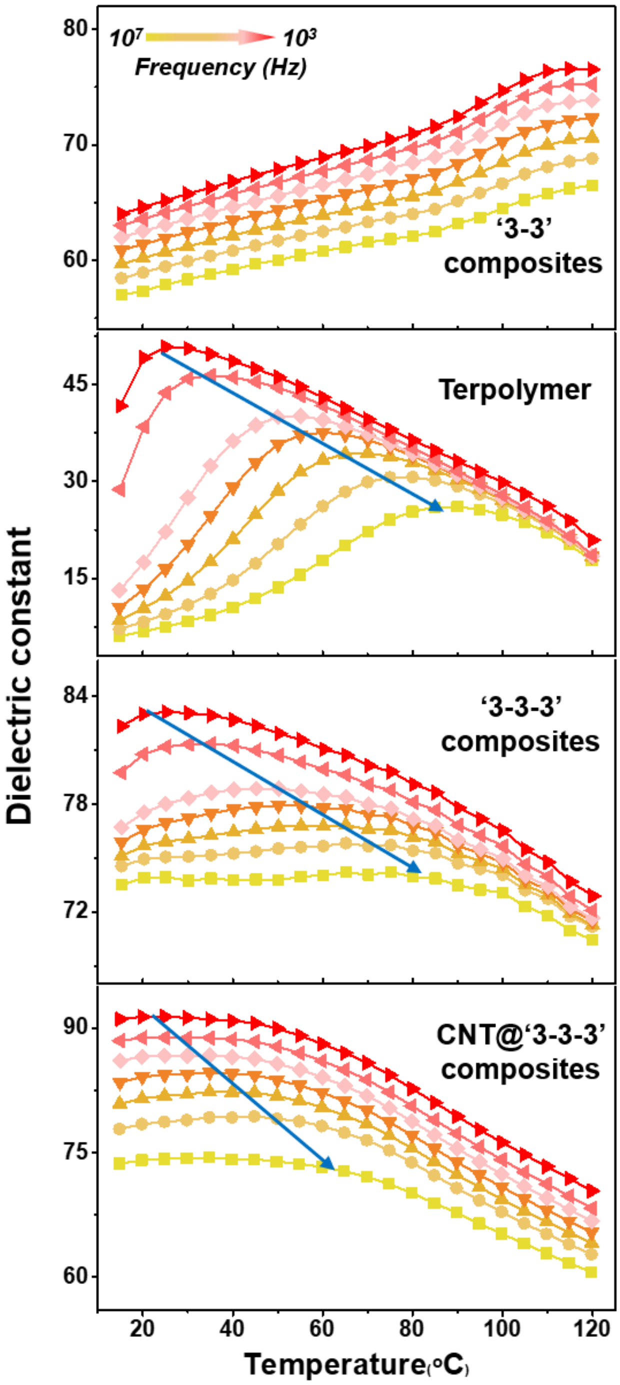

Fig. S4 Temperature-dependent dielectric spectra of '3-3' composite, terpolymer,

'3-3-3' composite and CNT@'3-3-3' composite. The temperature in the measurement ranges from 15°C to 120°C and the dielectric constant of the samples are measured at an interval of 5°C. Frequency ranges from 1.1 kHz to 10 MHz in the measurements. The arrows indicate the increase of frequency. The volume fraction of PZT skeleton in all the composites are ~14 vol%.

Determination of content of CNT-terpolymer in composites

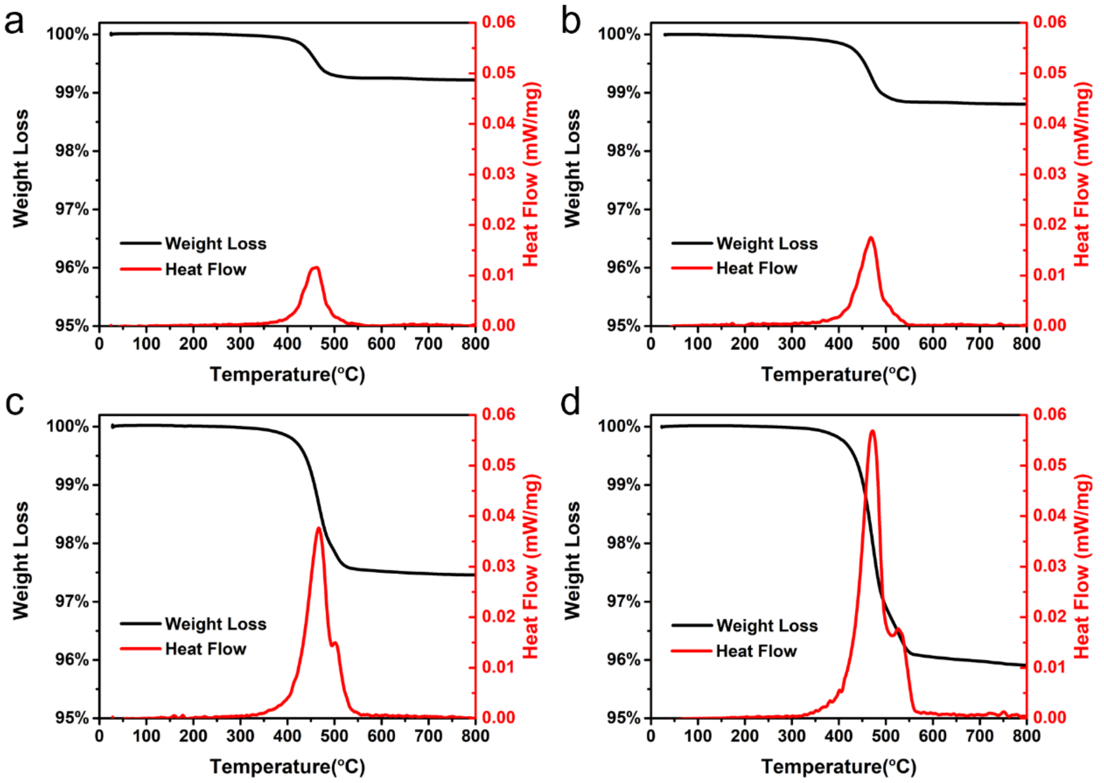

Fig. S5 Thermogravimetric analysis (TGA) of PZT skeleton coated with CNT-terpolymer layer. Four results correspond to PZT skeleton treated with polymer solution with different concentration. Terpolymer concentration in DMF was set to be (**a**) 2/15 w/v, (**b**) 4/15 w/v, (**c**) 6/15 w/v and (**d**) 8/15 w/v. The '3-3-3' composites used for dielectric and piezoelectric characterization are all treated with solution with polymer concentration of 6/15 w/v. It is worth noting that the mass fraction of terpolymer on PZT skeleton obtained from TGA analysis is not the final content of terpolymer in the composites which should be further calculated according to the PZT volume content in the composites.

Thermogravimetric analysis (TGA) is conducted to determine the content of CNT-terpolymer layer in '3-3-3' composites (Fig. S5). Samples used for TGA analysis can be obtained by coating sintered PZT skeleton with terpolymer layer followed by a drying process to exclude the solvent and water attached to the samples. Several samples treated by terpolymer solution mixed with CNTs with different concentration

are analyzed. With an increase in solution concentration, mass fraction of terpolymer layer coated on ceramic skeleton also increased. By calculating the mass fraction of terpolymer in ceramic skeleton, terpolymer content in final composites can be determined by further analyzing the content of terpolymer-coated ceramic skeleton in PDMS matrix. Generally, the maximum volume fraction of terpolymer in composites is ~1 vol% in samples treated by solution with terpolymer concentration of ~6/15 w/v (Fig. S5c) and the volume fraction of CNT in composites is ~0.01 vol%.

Electrical conductivity of composites with 3-3-3 connectivity

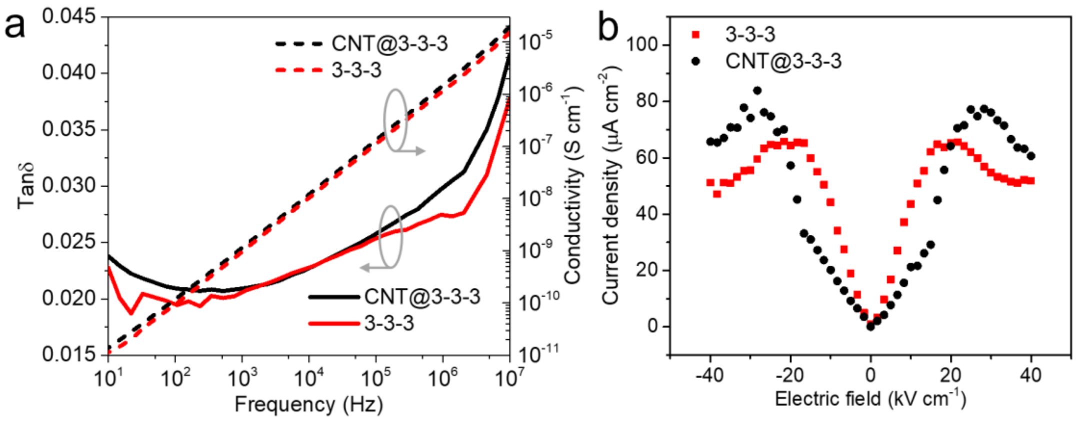

Fig. S6 (**a**) Frequency-dependent dielectric and conductivity spectrum. (**b**) dc leakage current. The PZT volume fraction in the composites are ~14 vol%.

The introduction of a very small amount of CNTs in the terpolymer layer doesn't induce noticeable increase in electrical conductivity and dielectric loss of CNT@3-3-3 composites as compared to the 3-3-3 composites without CNTs, as is shown in Fig. S6a. Besides, only a slight increase in dc conductivity under high dc electric field is observed for CNT@'3-3-3' composites according to measurement of dc leakage current (Fig. S6b).

Supplementary note 4 Simulation on dielectric and mechanical property of composites

Phase-field simulation on local electric field distribution

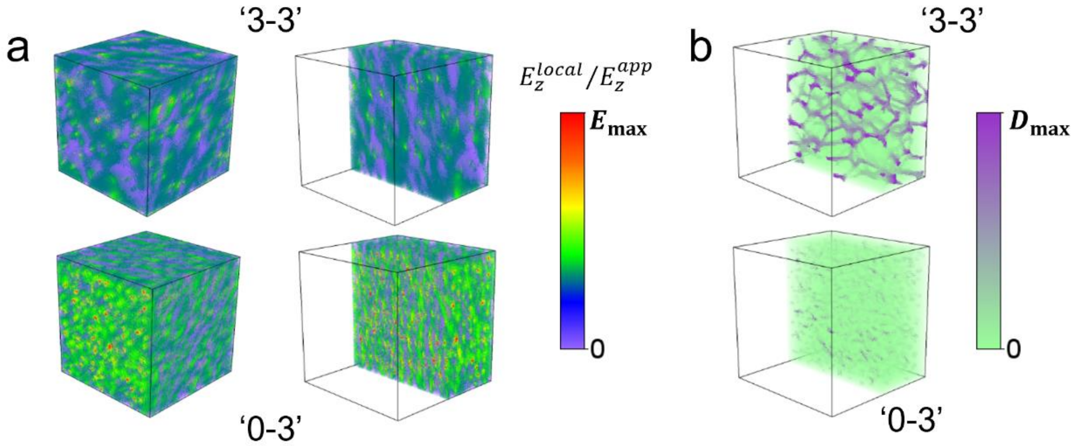

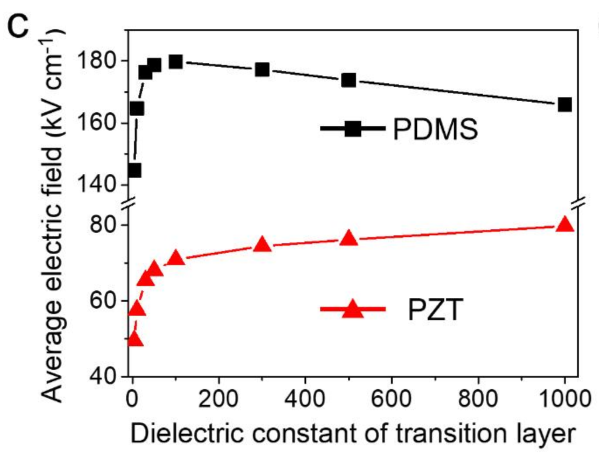

d

Dielectric constant of layer	Average electric field (kV cm^{-1})	
	PZT	PDMS
4	49.5	144.7
10	57.6	164.7
30	65.4	176.3
50	68.1	178.7
100	70.9	179.8
1000	79.8	165.9

Fig. S7 Phase-field simulation on local electric field distribution in '0-3', '3-3' and '3-3-3' type composites. Simulation on (**a**) normalized local electric field and (**b**) normalized local electric displacement distribution. The PZT content was set at 10 vol% for both '3-3' and '0-3' composites in the simulation. Normalized local electric field distribution of '3-3' composites (top of **a**) and '0-3' composites (bottom of **a**) and normalized local electric displacement distribution of '3-3' composites (top of **b**) and '0-3' composites (bottom of **b**) are displayed in the cubic model. (**c**), (**d**) Simulated average electric field in PZT and PDMS as a function of dielectric constant of the dielectric transition layer in '3-3-3' composite.

Phase-field simulation was conducted to illustrate the influence of filler arrangement on electric field distribution and polarization property of the composites. As is indicated by simulation results, the normalized electric field distribution in ‘3-3’ composites is more homogeneous than ‘0-3’ counterparts (Fig. S7a). The interconnection of ferroelectric filler reduces the local electric field concentration at the filler-matrix interface as a result of ease of depolarization field [5, 6]. This adjusted field distribution results in a dramatically enhanced electric displacement in PZT phase in ‘3-3’ composites (Fig. S7b), corresponding to a higher electric polarization.

We demonstrate that a further enhanced average electric field in PZT phase can be obtained by design of dielectric transition layer at PZT-PDMS interface. By assuming the dielectric constant of matrix, PZT and terpolymer layer to be 4, 1000 and 50, respectively, the average electric field in PZT is increased from 49.5 kV cm^{-1} to 79.8 kV cm^{-1} under applied electric field of 100 kV cm^{-1} (Fig. S7, c&d). In general, the local electric field in PZT increases with increasing dielectric constant of dielectric transition layer and gradually becomes saturated as the dielectric constant of the dielectric transition layer goes beyond 100. Besides, the average electric field in PDMS first increases and then decreases with increasing dielectric constant of the dielectric transition layer.

Polarization orientation and domain evolution under external electric field is found to significantly affect the electro-mechanical coupling performance of piezoelectric solid solution [7-9]. The contribution of polarization connection is also believed to greatly enhance the piezoelectric performance of ‘3-3-3’ composites. We used phase-field method to analyze the polarization and domain evolution in pristine PZT skeleton and skeleton coated with terpolymer layer. Polarization continuity of porous PZT skeleton is found to be improved with terpolymer layer covering the micropore area in ferroelectric skeleton structure (Fig. S8, a-f). The polarization intensity at different angles between polarization vector and <001> direction is also plotted for PZT

skeleton with and without terpolymer coating layer (Fig. S8g) and the total polarization vector along the direction of applied electric field is drastically enhanced for PZT skeleton coated with terpolymer layer.

Phase-field simulation on domain structure and polarization distribution

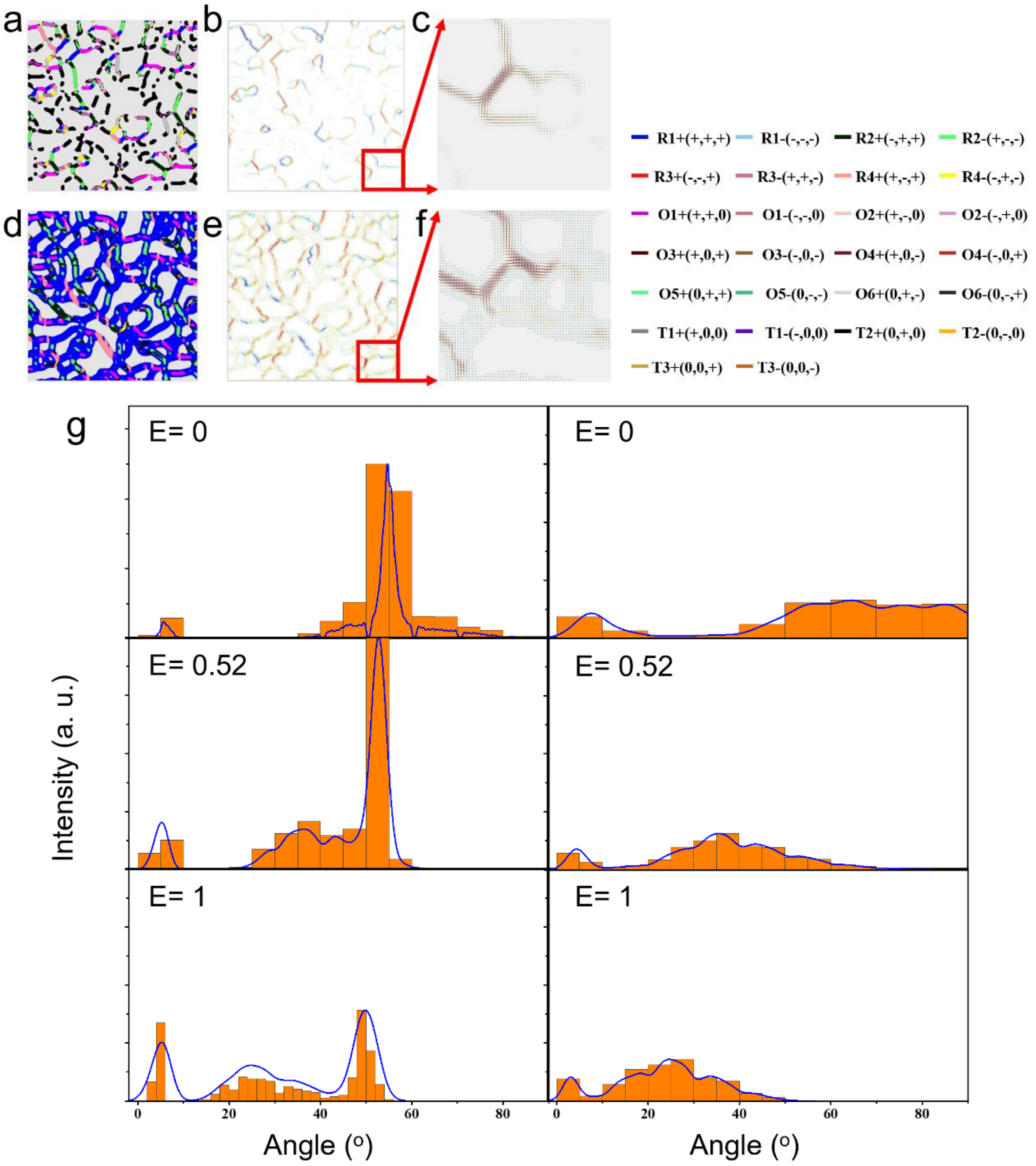

Fig. S8 Phase-field simulation on domain structure and polarization switching. Simulated domain structure of PZT skeleton (**a**) and polarization vector (**b** and **c**). (**d**)-(**f**) Simulated domain structure of PZT with terpolymer layer (**d**) and polarization vector (**e** and **f**). The figure legends to the right corresponds to the phase structure of the skeleton where the sign in the parentheses represents the direction of the polarization vector, i.e., (+, +, +) means the polarization vector lies in the first

quadrant. (**g**) Distribution of angle between polarization vector and poling direction (<001> direction) for PZT skeleton and PZT skeleton with terpolymer layer at different applied electric fields. The normalized external electric field for simulation was set to be 0, 0.52 and 1. The intensity for all figures is normalized.

Simulation on local strain/stress distribution

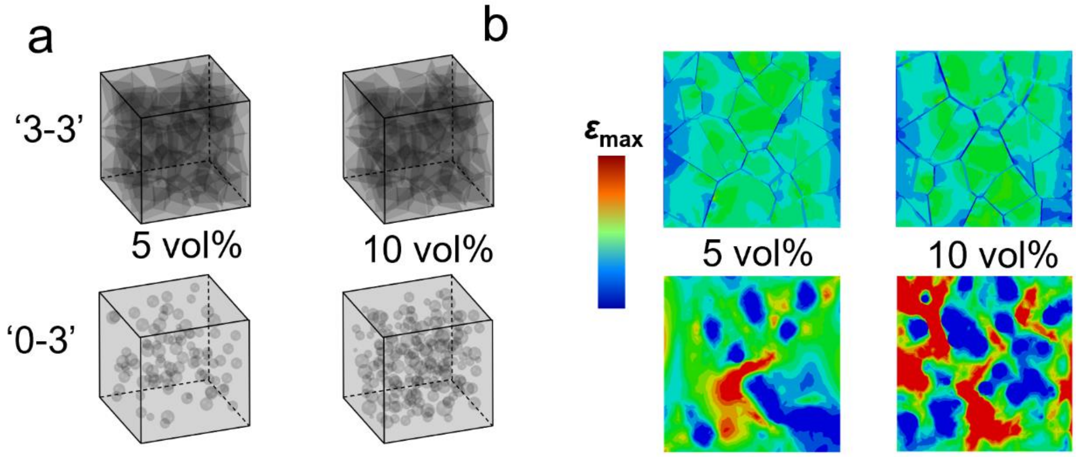

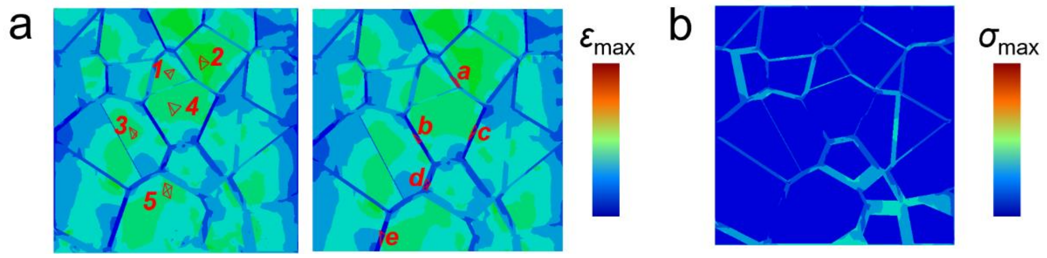

e

Data points of normalized local strain					
PDMS	1	2	3	4	5
	1.52	1.73	1.5	1.73	1.34
PZT	a	b	c	d	e
	0.23	0.18	0.2	0.16	0.19
The nominal strain of the composites is normalized to be 1.					

Fig. S9 Finite-element simulation of the local strain and stress distributions. (**a**) 3-D geometric models of '0-3' and '3-3' composites with PZT contents of 5 vol% and 10 vol%, respectively. (**b**) Corresponding simulated distribution of the local strain in composites with 5 vol% and 10 vol% PZT contents under uniaxial stretching (nominal

strain = 5%). (**c**) Simulated strain distribution in '3-3' composites with 15 vol% PZT content, under uniaxial stretching (5% nominal strain). The maximum local strain of five characteristic points marked in the skeleton and the matrix are adopted to calculate their average normalized values with nominal strain of composites set to be '1'. (**d**) Simulated distribution of maximum principal stress in '3-3' composites with 15 vol% PZT content, under uniaxial stretching (5% nominal strain). (**e**) Simulated normalized strain of the points marked in (**c**).

Supplementary note 5 Piezoelectric properties of composites

Rayleigh analysis of CNT@'3-3-3' composites

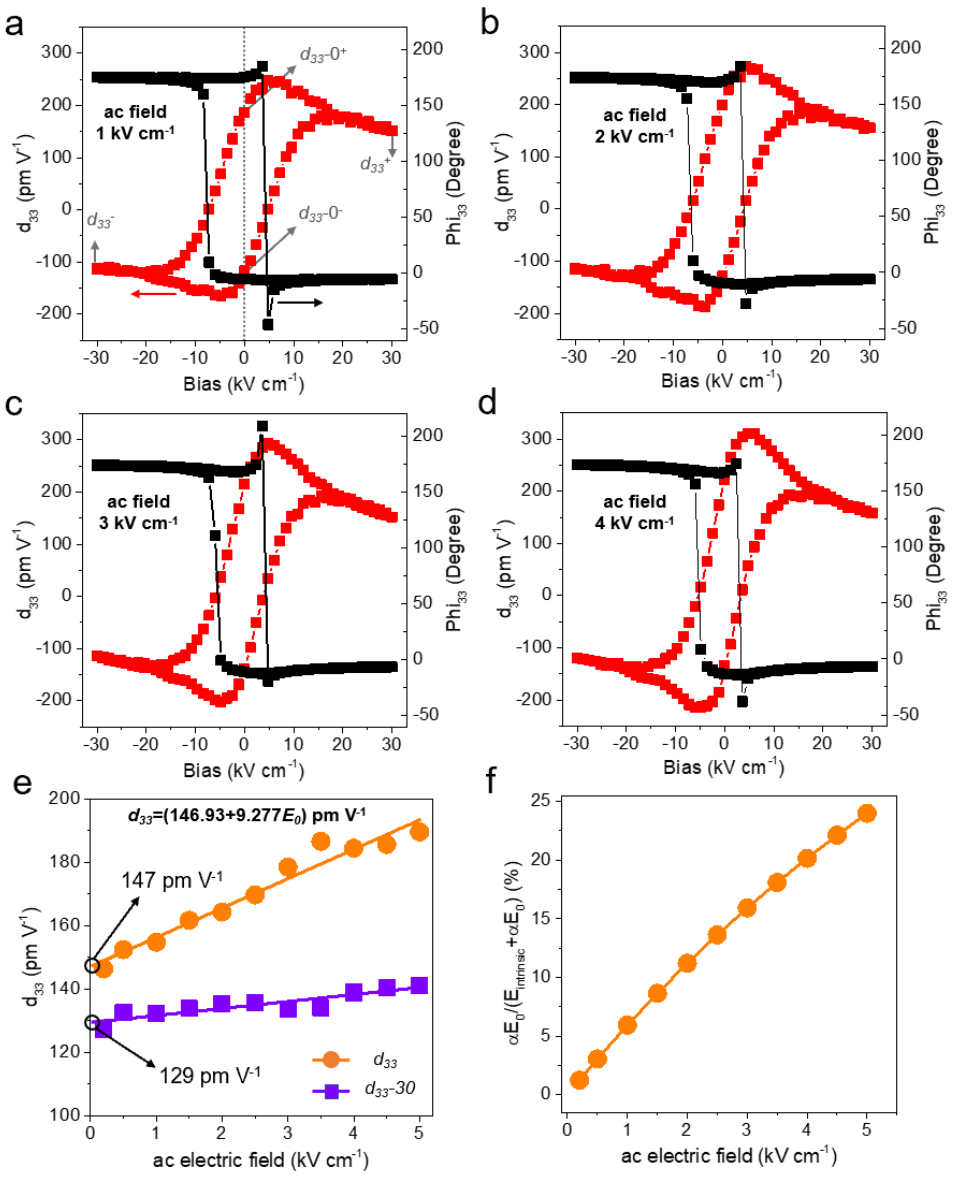

Fig. S10 Rayleigh analysis of CNT@'3-3-3' composite. (**a**)-(**d**), Small signal d_{33} measurement of CNT@3-3-3 composite with ~14 vol% PZT content. The maximum

dc bias is 30 kV cm^{-1} and the frequency of ac amplitude is 1 kHz for all. (**e**) ac electric field-dependent d_{33} for CNT@3-3-3 composite at 0 and 30 kV cm^{-1} dc bias. (**f**) $\alpha E_0/(\alpha E_0 + d_{intrinsic})$ of CNT@3-3-3 composite as a function of electric field.

We conduct Rayleigh analysis to further distinguish the intrinsic and extrinsic contributions to piezoelectric response of the studied composites. We measured the electric-field-dependent small signal piezoelectric coefficient for poled CNT@3-3-3 composites which is shown in Fig. S10. As shown in Fig. S10 a-d, an increase in applied dc bias leads to decreased piezoelectric coefficient of the composites which is similar to the electric-field-dependent piezoelectric response of PZT ceramic[1] as a result of the electrical clamping of domains in piezoelectric ceramic. As is shown in Fig. S10a, the small signal piezoelectric strain coefficient d_{33} at 0 dc bias is determined by the equation d_{33}=[d_{33}-0^+ – d_{33}-0^-)]/2 while the small signal piezoelectric strain coefficient d_{33} at 30 kV cm^{-1} dc bias is determined by d_{33}-30=[d_{33}^+ – d_{33}^-]/2. According to the Rayleigh law, the piezoelectric coefficient is dependent on ac electric field applied to the piezoelectric ceramic[2]. We plot the d_{33} values with variation of applied ac field in Fig. S10e.

The Rayleigh parameter *a* is 9.277×10^{-17} m^2/V^2 and the intrinsic response ($d_{intrinsic}$) of CNT@3-3-3 composite is 147 pm V^{-1} (orange data points) which is close to the piezoelectric charge coefficient (d_{33}=120 pC N^{-1}) measured by Berlincourt meter. The fitted piezoelectric response at 30 kV cm^{-1} dc bias (violet data points) decreased to 129 pm V^{-1} as a result of electrical clamping effect, suggesting a 12% domain wall contribution (147 pm V^{-1} to 129 pm V^{-1}) even at small signal measurement. The extrinsic contribution to the total piezoelectric response can be expressed as $\alpha E_0/(\alpha E_0 + d_{intrinsic})$ and is given in Figure. S10f. It is found that $\alpha E_0/(\alpha E_0 + d_{intrinsic})$ values show a progressive increase with the electric field increasing, being on the order of 24% at an ac electric field of 5 kV cm^{-1}.

<u>Finite-element analysis of piezoelectric performance</u>

We perform a series of finite-element modeling for the piezoelectric effects of our

samples via Abaqus. Numerical models are shown in Fig. S11a, composed of PZT skeleton and PDMS matrix with different volume ratios and length to thickness (L/H) ratios. Linear piezoelectric behavior was used for the modelling of PZT skeleton. The matrix [*c*] is the stiffness matrix, [*d*] is the piezoelectric strain matrix and the [*ε*] is the dielectric constant matrix.

$$[c]=\begin{bmatrix} 129060 & 83058 & 81461 & 0 & 0 & 0 \\ & 129060 & 81461 & 0 & 0 & 0 \\ & & 115760 & 0 & 0 & 0 \\ & & & 21053 & 0 & 0 \\ & & & & 21053 & 0 \\ & & & & & 23000 \end{bmatrix} \text{MPa}$$

(9)

$$[d]=\begin{bmatrix} 0 & 0 & -385 \\ 0 & 0 & -385 \\ 0 & 0 & 950 \\ 0 & 700 & 0 \\ 700 & 0 & 0 \\ 0 & 0 & 0 \end{bmatrix} \text{pC/N}$$

(10)

$$[\varepsilon]=\begin{bmatrix} 24.79 & 0 & 0 \\ 0 & 24.79 & 0 \\ 0 & 0 & 7.35 \end{bmatrix} \text{nF/m}$$

(11)

All samples are modeled by using 4-node tetrahedral solid elements (C3D4 in Abaqus). To calculate the piezoelectric strain coefficients, a potential difference is applied to the top and bottom of the simulated model. The strain per unit electric field in 3- direction (poling direction) and 1- or 2-direction denotes d_{33} and d_{31}, respectively. To compare the effect of matrix modulus to piezoelectric strain coefficients, a model with epoxy matrix (Young's modulus was 4 GPa) is also simulated. According to the simulation results, the transverse piezoelectric coefficient (d_{31}) always decreases

significantly faster than d_{33} with increasing porosity in all these models (Fig. S11b), indicating a suppressed lateral coupling in piezoelectric composites. The cuboid model with the defect structure has the absolute piezoelectric coefficient closest to the experimental value since the model has a most similar structure with the hierarchical porous structure in the samples. The modulus of the matrix material is also found to affect the piezoelectric coefficient of composites (Fig. S11c). Since PDMS used in experiment has a relatively small modulus below 1 MPa, the lateral coupling in piezoelectric composites is further minimized which contributes a higher d_h value [10].

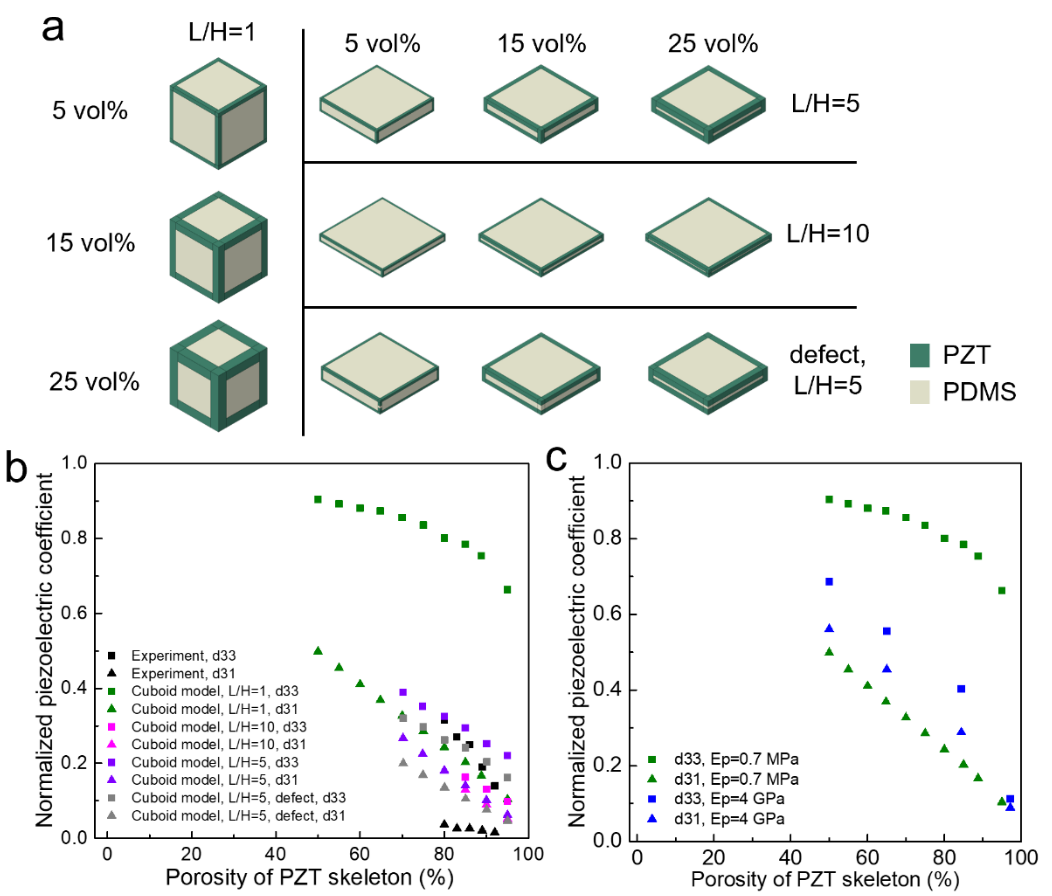

Fig. S11 Finite-element simulation of piezoelectric coefficients of the composites. (**a**) 3-D cuboid model of '3-3' type composites for finite-element simulation. (**b**) Experimental and simulated d_{33} and d_{31} of composites using different models. Normalized coefficients are defined as $d_{composites}/d_{ceramic}$ where $d_{composites}$ and $d_{ceramic}$ represent piezoelectric coefficient of composites and PZT ceramic, respectively. (**c**)

Simulated d_{33} and d_{31} of composites with matrix having different modulus. The Young's modulus of PDMS and epoxy resin are set to be 0.7 MPa and 4 GPa, respectively.

Piezoelectric coefficients and electro-mechanical coupling performance

We measured the thickness mode electromechanical coupling factor k_t of the CNT@'3-3-3' composite (~1 mm in thickness and ~10 mm in diameter) following the *IEEE* standards on piezoelectricity (ANSI/IEEE Std. 176-1987, *IEEE* Standard on Piezoelectricity) based on the measured resonant frequency (Fig. S12c and d) (f_r) and anti-resonant frequency (f_a) as:

$$k_t^2 = \frac{\pi}{2}\frac{f_r}{f_a}\tan\left(\frac{\pi}{2}\frac{f_a - f_r}{f_a}\right) \quad (12)$$

The high electromechanical coupling factor of 0.65 is very close to the electromechanical coupling factor of PZT ceramics in longitudinal mode (~0.75) but far beyond the thickness mode (~0.5).

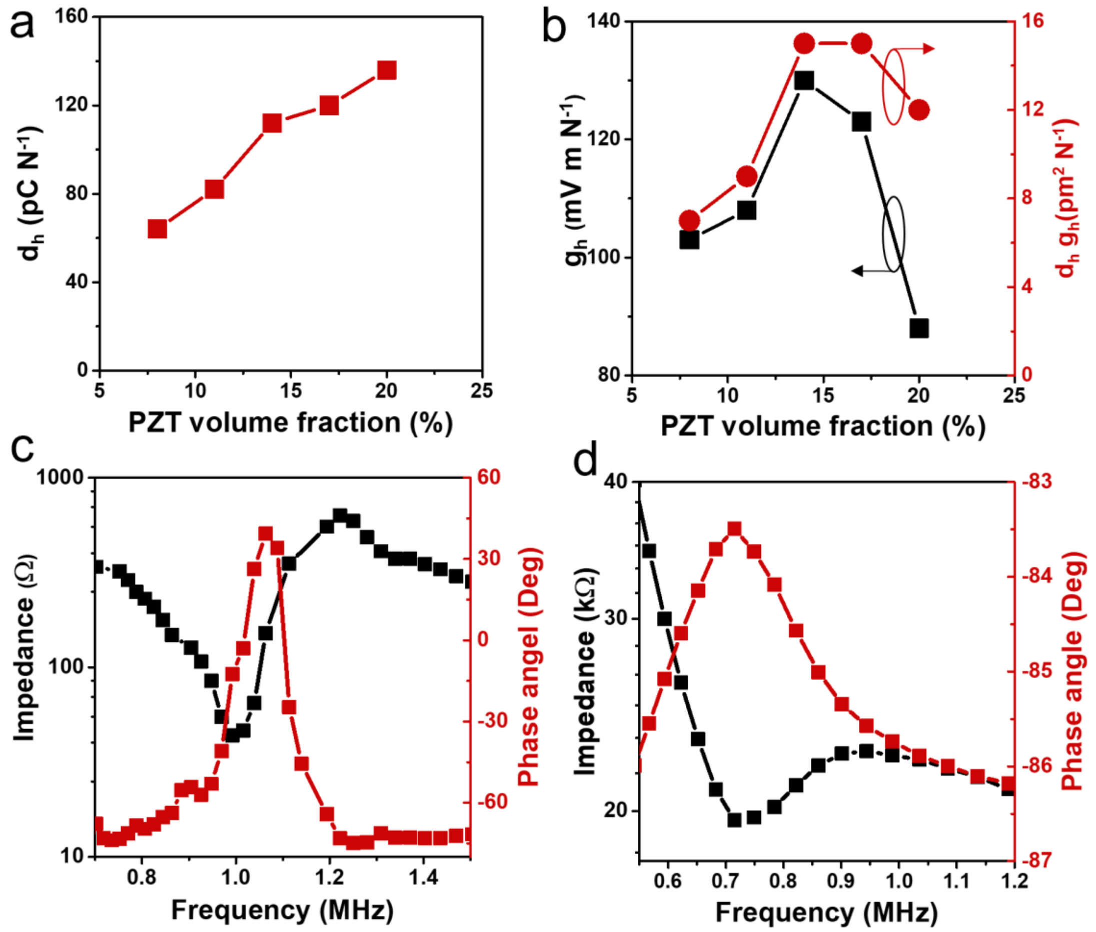

Fig. S12 Piezoelectric figure of merits of CNTs@'3-3-3' composites. (**a**) Experimental results of d_h values of CNT@'3-3-3' composites as a function of PZT content. (**b**) Experimental results of g_h and $d_h \cdot g_h$ values of CNT@'3-3-3' composites as a function of PZT content. (**c**), (**d**) Impedance spectrum and corresponding phase angle of PZT ceramic (**c**) and CNTs@'3-3-3' composites (**d**). The thickness of the PZT ceramic and CNT@'3-3-3' composites are ~ 1.1 mm.

<u>**Supplementary note 6** Characterization of composite-based transducer devices</u>

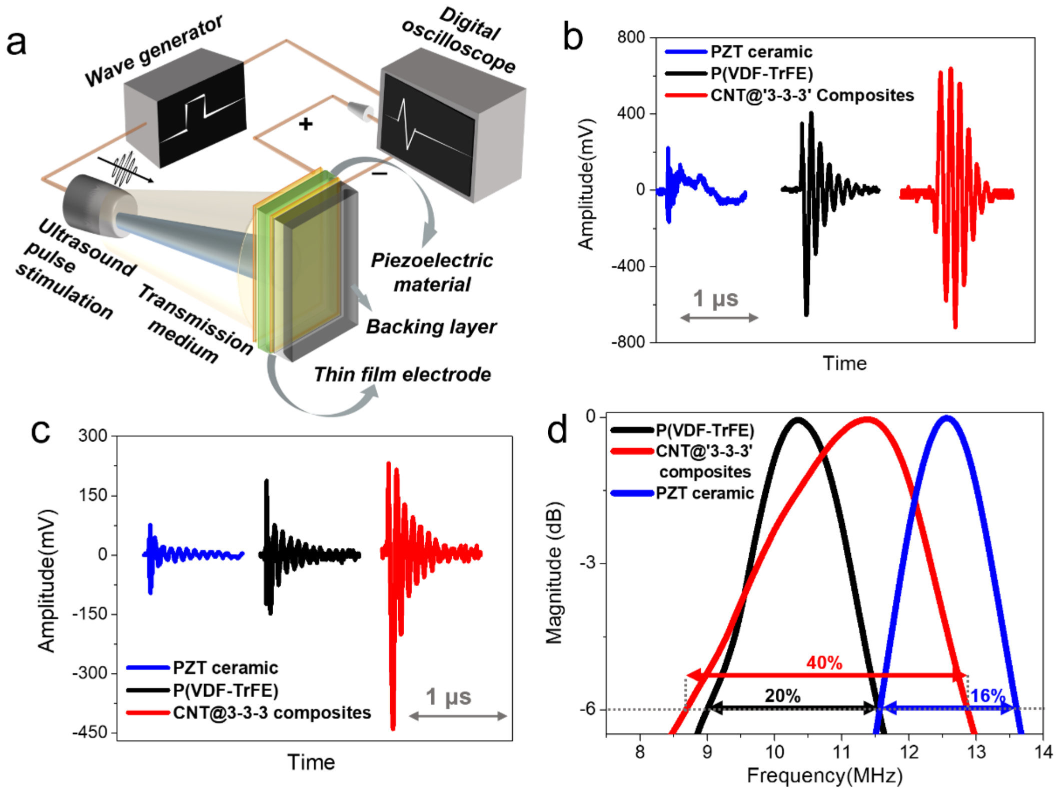

Fig. S13 Characterization of prototype ultrasonic transducers based on different piezoelectric materials. (**a**) Schematic diagram of the set-up for ultrasonic transducer measurement. (**b**), (**c**) Pulse amplitude of transducers based on PZT ceramic, P(VDF-TrFE) and CNT@‘3-3-3’ composites using coupling agent (**b**) and water (**c**) as the ultrasonic wave transmission medium. (**d**) Frequency-domain responses of transducers with normalized magnitude. The measurement was conducted using water as ultrasonic wave transmission medium. The -6 dB bandwidths of P(VDF-TrFE), CNT@‘3-3-3’ composites and PZT ceramic are 20%, 40% and 16%, respectively. All the transducers in experiment are free of acoustic matching layer. The thickness of the PZT ceramic and CNT@‘3-3-3’ composites are controlled to be ~ 170 μm and the thickness of P(VDF-TrFE) film is ~ 120 μm.

We demonstrate the excellent piezoelectric performance of the CNT@‘3-3-3’ composites by testing the pulse amplitude and frequency-domain responses of the composite-based transducers (Fig. S13) and the stability of output performance of the ultrasonic transducer based on the composites is analyzed by *in-situ* measuring the output voltage and frequency-domain characteristics under 20% compressive strain (Fig. S14). As seen, there is no decrease in output amplitude when the composite is subjected to 20% compressive strain, while the shift of center frequency from ~8

MHz to ~10 MHz can be associated with the decrease of composite thickness under compression.

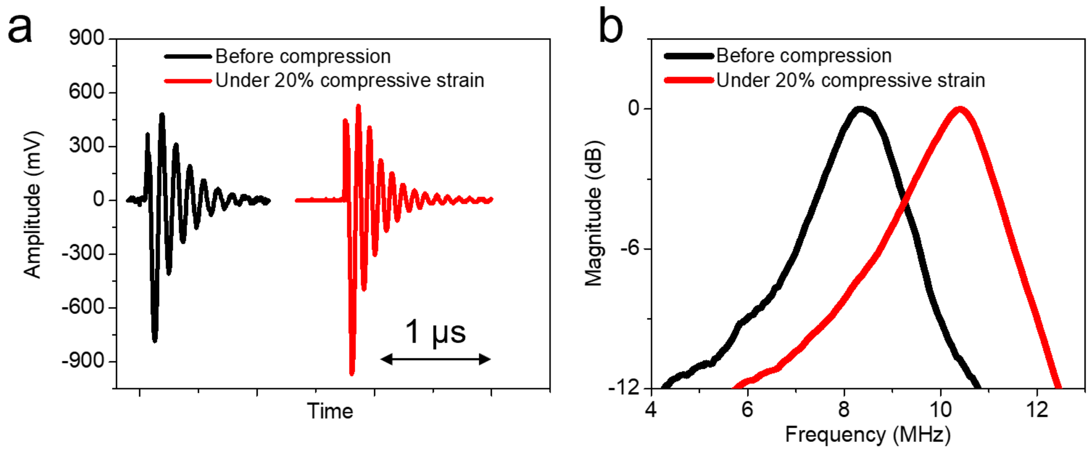

Fig. S14 Output performance of composite-based device under deformation. Output amplitude (**a**) and frequency-domain property (**b**) of composite-based transducer without and with 20% compressive strain. (The diameter of the composite is 10 mm and the applied dynamic force is kept at 40 N.).

Supplementary note 7 Characterization of mechanical properties

Macroscopic and *in-situ* deformation tests of CNTs@‘3-3-3’ composites

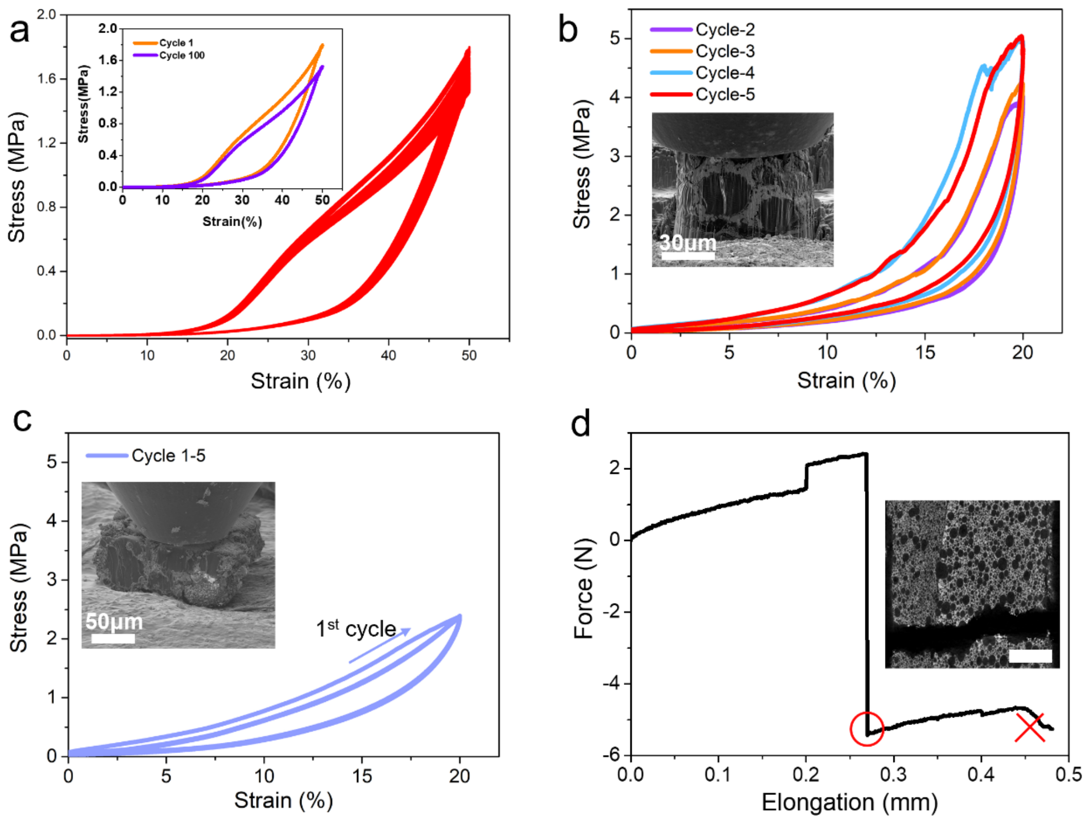

Fig. S15 Mechanical test on CNT@'3-3-3' composites. (**a**) Stress-strain curves during macroscopic compression test on the composite. The composite with ~ 14 vol.% PZT content was compressed for 100 cycles. (**b**), (**c**) *In-situ* compression test and the corresponding stress-strain curves for composites with 14 vol.% (**b**) and 10 vol.% PZT contents (**c**). (**d**) *In-situ* tensile test on composites. Force-elongation curve (**d**) and Micro-CT image of composites stretched to failure (inset figure). The scale bar in the inset figure is 500 μm. The abrupt jump marked by the red cycle in the curve (**d**) is due to the restart of tensile test after the scanning process. The composite finally fractured at ~0.48 mm elongation with a crack observed across the sample (inset figure) which corresponds to the red cross in the force-elongation curve (**d**).

Reference

1. Liu W, Xu J and Wang YZ *et al*. Processing and properties of porous PZT

ceramics from particle-stabilized foams via gel casting. *J Am Ceram Soc* 2013; **96**: 1827-31.

2. Mercadelli E, Sanson A and Pinasco P *et al*. Influence of carbon black on slurry compositions for tape cast porous piezoelectric ceramics. *Ceram Int* 2011; **37**: 2143-9.

3. Liu JJ, Li YB and Li YW *et al*. Effects of pore structure on thermal conductivity and strength of alumina porous ceramics using carbon black as pore-forming agent. *Ceram Int* 2016; **42**: 8221-8.

4. Huang HB, Zhang GZ and Ma XQ *et al*. Size effects of electrocaloric cooling in ferroelectric nanowires. *J Am Ceram Soc* 2018; **101**: 1566-75.

5. Ma FD and Wang YU. Depolarization field effect on dielectric and piezoelectric properties of particulate ferroelectric ceramic-polymer composites. *J Appl Phys* 2015; **117**: 124101.

6. Tomer V, Randall CA and Polizos G *et al*. High- and low-field dielectric characteristics of dielectrophoretically aligned ceramic/polymer nanocomposites. *J Appl Phys* 2008; **103**: 034115.

7. Fu HX and Cohen RE. Polarization rotation mechanism for ultrahigh electromechanical response in single-crystal piezoelectrics. *Nature* 2000; **403**: 281.

8. Li FX and Rajapakse R. Analytical saturated domain orientation textures and electromechanical properties of ferroelectric ceramics due to electric/mechanical poling. *J Appl Phys* 2007; **101**: 054110.

9. Picinin A, Lente MH and Eiras JA *et al*. Theoretical and experimental investigations of polarization switching in ferroelectric materials. *Phys Rev B* 2004; **69**: 064117.

10. Rittenmyer K, Shrout T and Schulze WA *et al*. Piezoelectric 3-3 composites.

Ferroelectrics 1982; **41**: 189.
